# Modelling optimal behavioural strategies in structured populations using a novel theoretical framework

**DOI:** 10.1038/s41598-019-51310-w

**Published:** 2019-10-21

**Authors:** Andrew Morozov, Oleg A. Kuzenkov, Elena G. Arashkevich

**Affiliations:** 1 University of Leicester, Department of Mathematics, Leicester, LE1 7RH UK; 2 N.I. Lobachevsky State University of Nizhny Novgorod, Nizhny Novgorod, Russia; 3 Shirshov Institute of Oceanology, Russian Academy of Sciences, 36, Nakhimovskiy prospect, Moscow, Russia

**Keywords:** Ecological modelling, Applied mathematics

## Abstract

Understanding complex behavioural patterns of organisms observed in nature can be facilitated using mathematical modelling. The conventional paradigm in animal behavior modelling consists of maximisation of some evolutionary fitness function. However, the definition of fitness of an organism or population is generally subjective, and using different criteria can lead us to contradictory model predictions regarding optimal behaviour. Moreover, structuring of natural populations in terms of individual size or developmental stage creates an extra challenge for theoretical modelling. Here we revisit and formalise the definition of evolutionary fitness to describe long-term selection of strategies in deterministic self-replicating systems for generic modelling settings which involve an arbitrary function space of inherited strategies. Then we show how optimal behavioural strategies can be obtained for different developmental stages in a generic von-Foerster stage-structured population model with an arbitrary mortality term. We implement our theoretical framework to explore patterns of optimal diel vertical migration (DVM) of two dominant zooplankton species in the north-eastern Black Sea. We parameterise the model using 7 years of empirical data from 2007-2014 and show that the observed DVM can be explained as the result of a trade-off between depth-dependent metabolic costs for grazers, anoxia zones, available food, and visual predation.

## Introduction

The complex behavioral responses and sophisticated life traits of organisms which are observed in the natural world are often considered to be outcomes of long-term evolutionary processes, and their quantitative description via mathematical modelling is usually challenging. Moreover, it is intuitively clear that optimal behaviour and/or life traits of an organism should gradually alter with maturation and progression through different developmental stages: a successful behavioral strategy for juveniles may not be effective for adults, which often experience a different environment. Structuring of populations in terms of size or developmental stage usually greatly enhances the complexity of our evolutionary models, and consequently existing modelling frameworks dealing with evolution in structured populations are somewhat less developed compared to those for unstructured populations^1,2^. The aim of this paper is to propose a framework to reveal optimal strategies for different developmental stages in stage-structured population models with continuous growth. Our study is based on a revisited concept of evolutionary fitness related to the long-term selection process and can be applied to deal with both scalar and function valued life traits or behaviours. As an important ecological study case, we explore patterns of regular diel vertical migration of zooplankton in aquatic ecosystems under variable environmental constraints.

The conventional wisdom of evolutionary modelling used to predict optimal life traits or behaviour is often based on the generic idea that a certain quantity known as the evolutionary fitness should be maximised^3–6^. However, the definition of fitness for an organism or a subpopulation is generally subjective and may depend on the personal preference of the researcher. For example, the existing models of diel vertical migration of zooplankton use different definitions of population fitness such as the individual reproductive value^7,8^, the inverse mortality^9,10^, the ratio between the food intake and the mortality^11–13^, and the ‘venturous revenue’^14^ among others. As an unfortunate result, models of optimal behaviour using different definitions of fitness can predict clearly distinct patterns^14–16^. In recent works, however, a new mathematically straightforward approach for identifying the evolutionary fitness has been proposed^16–18^. This approach considers long-term dynamics of competing subpopulations which are described by different inherited units (e.g. behavioural strategies, life traits, genotypes). The evolutionary fitness can be defined based on the comparative ranking order of such subpopulations. As a result, the long-term evolutionary outcome only depends on the choice of the underlying population model rather than a speculative metric of fitness^16^.

Here, we extend the ideas of the approach mentioned above^16,17^ to explore optimal behavioral patterns in a generic age- or size-structured population model with continuous age or size settings. In particular, we demonstrate how the evolutionary fitness can be derived for a generic von Foerster model with an arbitrary mortality term. Then we show that the optimal patterns of behaviour which are observed in the model can be derived by applying the variational principle of natural selection. Using this principle we obtain equations providing the optimal trajectories of regular vertical migration of zooplankton.

Regular diel vertical migration (DVM) of marine and freshwater zooplankton is often regarded as the largest synchronised movement of biomass on Earth^19,20^. Understanding DVM is so important because it plays a key role in the carbon exchange between the deep and surface waters, the oceans biological pump^21,22^. Typically, DVM consists of planktonic grazers ascending to plankton-rich surface waters to feed at night, then descending to deeper waters and remaining there during the day^19,23^. There exist several explanations of what causes organisms to migrate, but the most accepted hypothesis is currently that zooplankton perform DVM to avoid visual predation, mostly by planktivorous fish, by staying in the deeper and darker areas during daylight hours and ascending at night when visual predators are unable to see them^23–26^. The DVM phenomenon has been extensively studied both empirically^23–26^ and theoretically using a number of mathematical models^8,26–28^. However, we should stress that most of the existing models of DVM were based on the principle of maximization of some initially prescribed fitness criterion which was a personal choice of researcher, thus the generality of the modelling results remains questionable. Furthermore, the role of key factors affecting DVM is still poorly understood. This particularly concerns the difference in migration behaviour for different developmental stages and the influence of environmental factors such as food, temperature, oxygen, and predators on the amplitude and timing of migration for each stage. We argue that mathematical modelling based on our revisited concept of fitness and also backed up by long-term empirical observation can provide us with a better understanding of zooplankton DVM^7,13,27,29,30^.

Here we implement the novel theoretical framework to explore the DMV patterns of dominant zooplankton herbivores in the north-eastern Black Sea ecosystem. We differentiate between strategies of different zooplankton developmental stages and consider how key environmental parameters such as food availability, predation, and habitat size affect the optimal DVM. Using the model, we address the long-standing question of why the migration depth of zooplankton is two-three times greater than the size of the euphotic zone^31–33^. Our modelling research into optimal DVM is backed up by 7 years of empirical observation (2007–2014) of the migration of two abundant copepod species, *Calanus euxinus* and *Pseudocalanus elongates*, across seasons and under different biotic and abiotic conditions. Our study demonstrates that it is the depth-dependent variability of the metabolic costs of the grazers - related to the specific oxygen regime in the Black Sea - rather than trophic pressure by visual predators that determines the choice of the lowest migration depth. The model also predicts that the absence of diel migration of earlier stages of zooplankton might be mostly due to their high mortality rather than due to a high cost of DVM, contrary to what was believed previously.

The paper is organized as follows. Section 2.1 introduces the generalized variational principle of modelling natural selection and introduces the generalised fitness function. In Section 2.2, a generic size- or age- structured model is introduced and the evolutionary fitness for this model is derived. In Section 2.3, the generic model is implemented to explore patterns of optimal zooplankton DVM. In particular, Subsection 2.3.1 provides empirical evidence for zooplankton DVM in the Black Sea; subsections 2.3.2 explain parameterisation of the model coefficients; Subsection 2.3.3 shows the modelling results on the optimal DVM. The discussion in Section 3 summarises the main results and provides further ideas on revealing optimal behavioral strategies using the new framework.

## Results

### Establishing general variational principles of natural selection

Here we provide a generic mathematical framework of modelling natural selection in a self-replicating system with inheritance and introduce a mathematically rigorous definition of evolutionary fitness.

Consider some population, where organisms are described by inherited elements *v*, strategies or life traits, for instance. Mathematically, an element *v* can be a scalar, a vector, or a function, so we consider that *v* belong to a certain function space *V*. In particular, for a stage-structured population, *v* can be a vector of functions describing each developmental stage. From now on we will refer to *v* as a strategy for the sake of simplicity. For simplicity, we consider that strategy *v* is passed unchanged to each offspring from its parent, i.e. as in the case of a clonal reproduction (note that our methodology can be extended to a more complicated case allowing for mutations^16^). We assume that *v* belongs to a compact domain in a metric space *V* equipped with a Borel measure μ*. We also assume that the measure of any open set (except $\varnothing$) is greater than zero.The need for introducing a metric and using a measure comes from the following.

We assume that elements of *V* can be somehow compared in terms of similarity (or closeness) to each other. Mathematically, this means that we need to introduce a distance between elements *v*; thus the space *V* is required to be a metric space. The existence of a metric allows us to generate neighbourhoods and open sets in *V*. Our framework should also be able to quantitatively describe and compare the sizes of different sets in *V*. For this purpose, we use the concept of *σ* - algebra of ∑ subsets in *V* and then introduce a measure of those subsets. Note that almost for any choice of subsets ∑, there will be always non-measurable sets. Thus, it is logical to use only such *σ* - algebra which will contain all subsets which are of interest for further applications (e.g. all open sets). The minimal *σ* - algebra containing open sets is known as a Borel algebra, and a measure introduced on it is called a Borel measure. Note that in practical applications, almost all considered measures are Borel measures. For examples, in the finite dimension space, a Borel measure will be extension of the ‘classical’ concept of volume. In infinite dimension spaces (function spaces), a Gaussian measure is typically used^34^.

The presence of the subpopulation with strategy *v* at time *t* is indicated by a non-negative numeric value $\eta (v,t)$ which can be understood as a generalized density. This may be the density of the subpopulation with strategy *v*, but we can also describe the presence of strategies in the population via the logarithmic scale of the biomass, for example. Alternatively, we can characterize the presence of strategies in the population via any positive power of population size which can be strategy-dependent. We postulate that $\eta (v,t)$ should satisfy the following requirements:

- $\eta (v,t)=0$ indicates the absence of *v* in the population at time *t*.
- $\eta (v,t) > 0$ indicates the presence of *v* in the population at time *t*.
- If $\eta (v,t)$ approaches zero this signifies extinction of *v*.
- It is a continuous function over the space *V* and it is integrable over *V* with regard to μ*.
- It is a smooth function of time.
- ${\int }_{V}\eta (v,t){\mu }^{\ast }(dv)$ is uniformly bounded by a constant, i.e. ${\int }_{V}\eta (v,t){\mu }^{\ast }(dv)\le c$ for any *t*. This takes into account the natural assumption that limitation of resources for the population restricts its population growth.

We suggest that we know the equation which govern the temporal dynamics of $\eta (v,t)$; for example, it can be derived from the underlying model of population dynamics.

We can now introduce the following definition of ranking of strategies.

#### Definition 1

(**Ranking order of strategies**). We state that element *v*′ is better (or fitter) than element *w*′ ($v^{\prime} \succ w^{\prime}$), if there exists a neighborhood $O(v^{\prime} )$ of point *v*′ and a neighborhood $O(w^{\prime} )$ of point *w*′ such that the ratio of generalised densities (1) tends to zero uniformly in the neighborhoods $O(v^{\prime} )$ and $O(w^{\prime} )$ for *v* in the neighborhood $O(v^{\prime} )$ and *w* in $O(w^{\prime} )$, i.e.

1 $$\frac{\eta (w,t)}{\eta (v,t)}\mathop{\to }\limits_{uniformly}0,t\to \infty .$$

Note that the above definition should be considered as a partial ordering and may also depend on the initial conditions in the system. Using this definition of the ranking order of two strategies one can easily conclude that the subpopulation with a strategy of lower ranking should eventually go extinct. This is formally given by the following theorem.

#### Theorem 2.

*If v*′ is a better strategy than *w*′ ($v^{\prime} \succ w^{\prime}$) then there exists a neighborhood $O(w^{\prime} )$ such that the generalised density $\eta (w,t)$ tends to zero with $t\to +\infty$ uniformly in $O(w^{\prime} )$.

The proof of the above theorem is given in Supplementary Material SM1(i). Using the above definition of ranking and the theorem, one can now define the evolutionary fitness in the system as follows.

#### Definition 3

(**Evolutionary fitness**). In the case where there exists a functional $J(v)$ such that it preserves the ranking order of strategies given by, i.e. $J(v) > J(w)\Rightarrow v\,\succ \,w$, this functional is referred to as an evolutionary fitness.

Maximizing a given fitness function provides the variational principle of modelling natural selection: only the strategies v* realizing the global maximum of the fitness *J* will remain in the population, the others will go extinct. Thus, the strategies v* will be evolutionary optimal. We should stress again that, since the ranking order for a particular population model may depend on the initial conditions, the evolutionary fitness can also depend on the initial conditions. Also, the fitness functional is not unique: any increasing function of *J* will also be an evolutionary fitness.

According to the formulated variational principle of selection, we need to find an evolutionary fitness based on model equations and then find the strategy or strategies which maximise its value. Note that in general, finding *J* can be challenging. In the next section we will show how the evolutionary fitness can be derived for an age- or size- structured population model described by the von Foerster equation.

### Deriving evolutionary fitness in an age (stage)-structured model

We consider a single population model with structuring described by a von-Foerster-type equation^35,36^. The population is characterised by the density $z(v,W,t)$ at the moment of time *t* with body weight *W* and behavioral strategy *v*. Here by *v* we understand the overall set of strategies across all developmental stages, where each strategy can be a function with known mathematical formulation with a fixed number of evolving parameters or can be an unspecified function-valued trait, essentially an infinite number of evolving parameters. The evolution is governed by:

2 $$\frac{\partial z(v,W,t)}{\partial t}+\frac{\partial z(v,W,t)r(v,W)}{\partial W}=-\,A(v,W)z(v,W,t)-R(v)z(v,W,t)y(t),$$

where $r(v,W)$ describes the increase in the body weight of individuals due to growth; $A(v,W)$ is the linear mortality rate. The second mortality term in the right hand side describes effects of competition between adults and juveniles across all possible strategies as well as possible effects of predation, harvesting, or any external forcing. Note that in this term, we incorporate the effects of strategy into *R*, and consider that $y(t)$ equally affects the mortality of each stage.

The production of offspring which initially have a minimal body weight ${W}_{{\min }}$ is due to the reproduction of the whole cohort of adults which is given by

3 $$z(v,{W}_{{\min }},t)r(v,{W}_{{\min }})={\int }_{{W}_{A}}^{+\infty }b(v,\theta )r(v,\theta )z(v,\theta ,t)d\theta ,$$

where $b(v,W)$ is the reproduction coefficient. Biologically, *W*_*A*_ is the weight at which organisms become mature and can reproduce, after which $b(v,W) > 0$. The increase of the body weight *W* is described by the following growth equation:

4 $$\frac{dW}{dt}=r(v,W),W(0)={W}_{{\min }},$$

where $r(v,W)$ is the body growth rate.

Model (2) with structuring in terms of body weight can be transformed^37^ to the equivalent model with structuring in terms of age *τ* by introducing the following variable $\rho (v,\tau ,t)=z(v,W,t)r(v,W)$. The equations for $\rho (v,\tau ,t)$ will read

5 $$\frac{\partial \rho (v,\tau ,t)}{\partial t}+\frac{\partial \rho (v,\tau ,t)}{\partial \tau }=-\,A(v,\tau )\rho (v,\tau ,t)-R(v)\rho (v,\tau ,t)y(t),$$

and the renewal equation becomes

6 $$\rho (t,0,v)={\int }_{{\tau }^{\ast }(v)}^{+\infty }b(v,\theta )\rho (v,\theta ,t)d\theta .$$

Here $\rho (v,\tau ,t)$ should be understood as a re-scaled population density; ${\tau }^{\ast }(v)$ is the time at which organisms mature and can begin to reproduce.

The weight *W* and the age *τ* are related by $W=W(v,\tau )$ (we assume that $r(v,W) > 0$ which should guarantee a one to one correspondence between *τ* and *W*), in particular, the maturation age τ* is given by ${W}_{A}=W(v,{\tau }^{\ast })$. For simplicity, we will further consider model (5) - (6) for discrete stages $i=0,1,\mathrm{...},n$. For each stage *i* and for a given strategy *v* we assume the model coefficients to be constant. Thus, for the mortality and production rates we have

$$A(v,\tau )=(\begin{array}{ccc}{a}_{0}(v), & 0\le \tau  < {\tau }_{1}, & \\ {a}_{i}(v), & {\tau }_{i}\le \tau  < {\tau }_{i+1}, & \,1\le i\le n-1\\ {a}_{n}(v), & {\tau }_{n}\le \tau  < +\,\infty , & \end{array}$$

$$b(v,\tau )=(\begin{array}{ccc}{b}_{i}(v), & {\tau }_{i}\le \tau  < {\tau }_{i+1}, & \,1\le i\le n-1\\ {b}_{n}(v), & {\tau }_{n}\le \tau  < {\tau }_{n+1}, & \end{array}$$

Here we formally assume the reproduction to be possible from stage $i=1$. However, the model still allows us to consider the case where some reproduction rates ${b}_{i}=0$ for $i > 0$. Note also that the reproduction of the final stage ends at the age ${\tau }_{n+1}$, whereas the organisms can live for longer.

The evolutionary fitness is in the above structured model given by the following theorem.

#### Theorem 4.

In the age-structured model (5)– (6) with discrete stages $i=0,1,\mathrm{...},n$, the strategy which maximises the evolutionary fitness will outcompete the other strategies. The fitness is defined by

$$J(v)=\frac{{\max }_{i}\Re ({\lambda }_{i}(v))}{R(v)},$$

where *λ*_*i*_ is the solution (the eigenvalue) of the characteristic equation provided in SM1(ii); $\Re$ denotes the real part of this eigenvalue.

The proof of the above theorem is given in supplemental Material SM1(ii).

In this paper, we will consider the case where the number of developmental stages is 3 (i.e. $i=0,1,2$) and the reproduction starts from the oldest stage only (i.e. ${b}_{1}=0$). We denote ${b}_{2}=b > 0$. In SM1(iii), it is shown that in this case the characteristic equation determining the fitness *J* is given by

7 $$J=\frac{b}{R}\exp (-\,{a}_{0}{\tau }_{1}-{a}_{1}({\tau }_{2}-{\tau }_{1}))[\exp (-{\tau }_{2}JR)-\exp (-{\tau }_{3}JR-{a}_{2}({\tau }_{3}-{\tau }_{2}))]-\frac{{a}_{2}}{R}.$$

Thus, to find the optimal strategies for all three stages $v=({v}_{1},{v}_{2},{v}_{3})$, one needs to maximise fitness given by (7). Note that in the given model the fitness does not depend on initial conditions.

### Study case: exploring optimal DVM of zooplankton

#### Empirical observation of DVM

We now use the theoretical framework introduced above to explore patterns of diel vertical migration (DVM) of herbivorous zooplankton. The modelling study is motivated by our long-term empirical observation of DVM of zooplankton in the north-eastern Black Sea. Samples were collected throughout all seasons in the years 2007–2014 for two of the most abundant herbivorous mesozooplankton species: *Calanus euxinus* and *Pseudocalanus elongatus*. Details on samples collection and data analysis are provided in the supplementary material (SM2) as well as in a satellite paper^38^.

**Figure 1:**
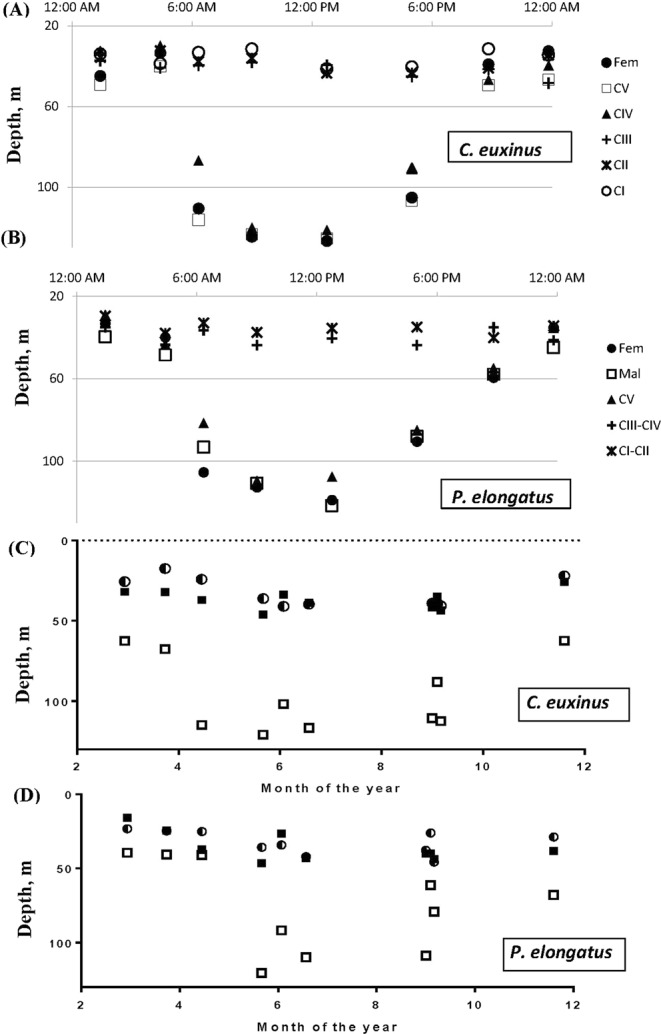
(**A**,**B**) Typical pattern of DVM of two dominant zooplankton herbivores *Calanus euxinus* and *Pseudocalanus elongatus* observed in the north-eastern Black Sea in summer. The samples were collected on 21/06/2011. For each developmental stage the centers of vertical abundance distribution is shown, see the main text and SM2 for detail. (**C**,**D**) Annual variation of the depths of DVM for migrating females (denoted by filled/open squares for night/day depths) and non-migrating stages CI-CIII (denoted by semi-filled circles).

Figure 1 show a typical pattern of DVM for the investigated species; the graphs represent the variation in the mean depths of the copepod distribution across the 24 h period for all developmental stages. Note that the zooplankton population is scattered around a certain depth in each instant, and the figures show the spatially average depth of the zooplankton distribution in the column (calculated using the computational algorithm from SM2). One can see that for both species only the copepods from the older developmental stages (starting from CIV) exhibit a pronounced migration: they feed on phytoplankon at depths of *h* = 35–45 m at night and stay in deeper waters (*h* = 110–120 m) during the daytime.

Interestingly, the amplitude of DVM for both species varies across seasons, as is demonstrated in Fig. 1C,D, which show the lower and upper depths for migrating females through the daily cycle (denoted by the open and filled squares, respectively) as well as the daily average depth of non-migrating stages of the same species (denoted by semi-filled circles). One can see that the deepest depth of DVM increases in summer and decreases in winter. In the literature, seasonal variation of migration depths is often connected to seasonal change of the width of (i) the oxygen zone in the north-eastern Black Sea and (ii) the zone with suitable water temperature ranges for the species considered^32^. However, the role of the oxygen zone and temperature conditions in DVM are not yet well understood and this model study is intended to shed some light on this long-standing question^31–33^.

**Figure 2:**
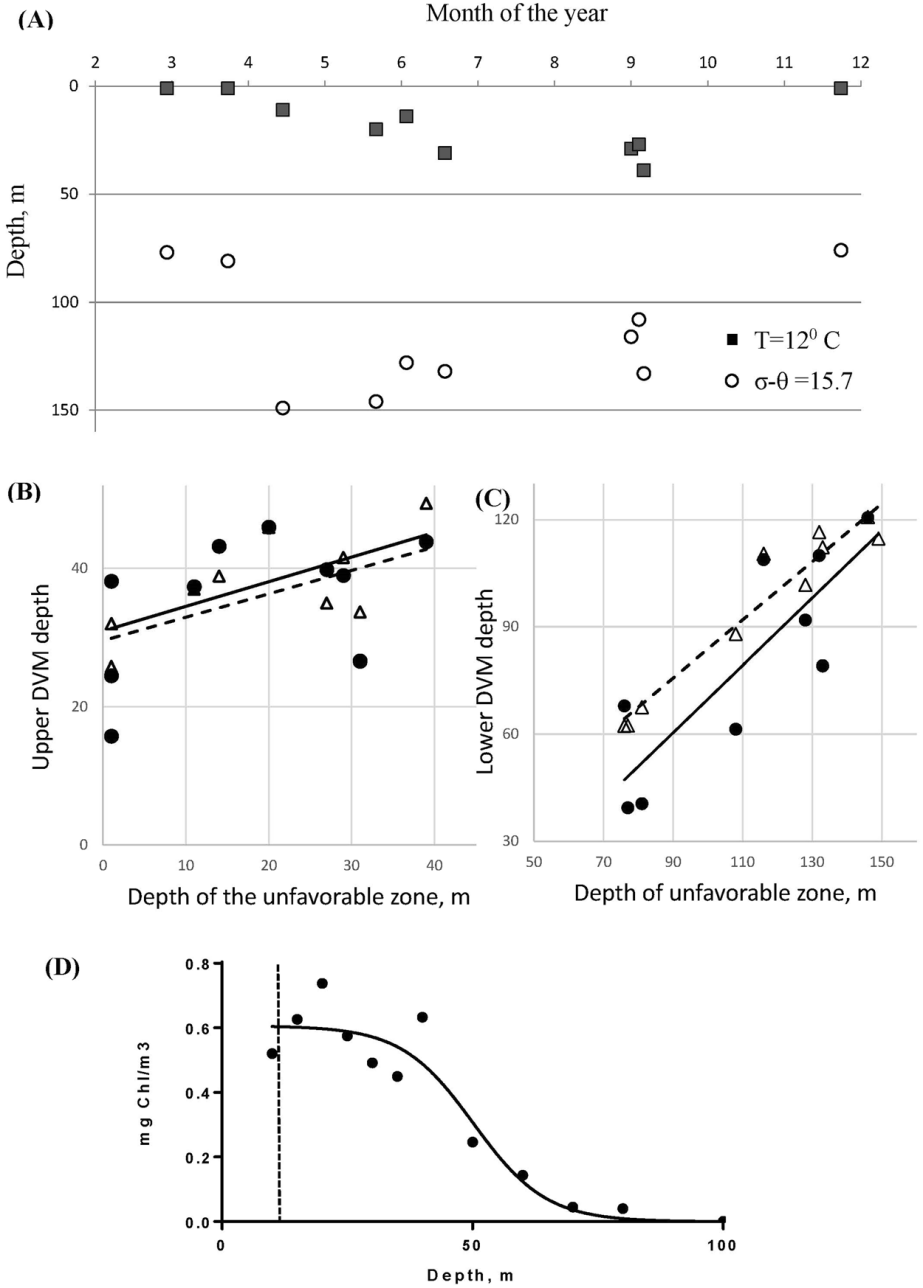
(**A**) Seasonal variation of the depths of the upper and lower unfavorable zones for *C. euxinus* and *P. elongatus* observed in the north-eastern Black Sea. The upper zone is dictated by temperatures higher than 12 °C, whereas the boundary of the lower zone is given by ${\sigma }_{\theta }$ curve achieving 15.7 (see SM3). Dependence of the upper (**B**) and the lower (**C**) depths of DVM (females) on the depths of the unfavorable zones corresponding to panel (**A**). Triangles and circles denote, respectively, *C. euxinus* and *P. elongatus*. The solid and the dashed lines fit, respectively, the data for *P. elongatus* and *C. euxinus*. (**D**) The average (across all observations) vertical profile of chlorophyll a. The curve fitting to the data is discussed in text.

The data confirms the link between the amplitude of DVM and the variation in the boundary of the oxygen and the temperate zone. Figure 2A shows the seasonal variation of the sigma-theta profile (its value of 15.7 approximately corresponds to the minimal comfortable oxygen concentration of 0.4 mg/l) as well as the depth of the temperature level of *T* = 12 °*C* which specifies the critical boundary for comfortable living for both species preferring colder temperatures. The influence of the oxygen level and the warm waters depths on DVM can be seen more clearly in Fig. 2B,C. Figure 2B demonstrates a positive correlation between the night time depths and the profile of the temperature level of *T* = 12 °*C* for both species (Pearson correlation coefficients are $r=0.860$ and $r=0.814$ for *C. euxinus* and *P. elongatus*, respectively). Figure 2 shows a stronger correlation between the maximal depth of migration and the density sigma-theta of 15.7 (Pearson correlation coefficients are $r=0.96$ and $r=0.841$, for *C. euxinus* and *P. elongatus*, respectively).

We also investigated the vertical profiles of Chl-a concentration as the index of autotrophic phytoplankton—the primary food for zooplankton grazers—in the water column. We found, however, that phytoplankton distribution is rather variable both seasonally and from year to year. For modelling purposes we combine the data on chlorophyll and construct the annual average density of chlorophyll using our observational data for 7 years (see Fig. 2D), and do not show here the chlorophyll profile for each year and season. We fit the data with the curve ${P}_{0}(\tanh (-\sigma (h-{h}_{p}))+1)/2$ which is further used in modelling; *h* is the depth (for details see the next section). This curve exhibits a poor fit in the surface waters, denoted by the dashed vertical line, but we can still implement the above fit for modelling purposes since individuals rarely enter the upper surface zone.

#### Parametrisation of the model

We further need to parameterise the model coefficients and relate them to the daily trajectory of the individual grazer in the water column, i.e. the vertical coordinate *h*(*t*) which describes the strategy *v* in our original model settings. We consider that *h* = 0 at the water surface and that the positive direction is downwards. We re-scale time so that a 24 h period corresponds to the interval $t\in [0,1]$, and assume that due to periodicity of this function we have $h(0)=h(1)$. Times *t* = 0 and *t* = 0.5 will correspond to midnight and midday, respectively. The life cycle of a zooplankton grazer consists of 6 distinct copepodite stages (see the previous section), however, here we combine some stages and only consider three age groups consisting of the youngest juvenile stages (CI-III), older juvenile stages (CIV-V) and reproductive adults (CVI). Their DVM patterns are mathematically described by the functions ${h}_{Y}(t)$, ${h}_{J}(t)$ and ${h}_{A}(t)$, respectively. Note that one can easily extend the approach to model all 6 developmental stages. For simplicity, we neglect the hatching time and consider $R(v)=const$ and consider that the reproduction time of adults is fixed ${\tau }_{3}-{\tau }_{2}={T}_{0}=const$.

The reproduction coefficient *b*(*v*) expressing the number of eggs produced by a female per day is given by

8 $$b(v)=\frac{1}{{W}_{0}}{\int }_{0}^{1}({\varepsilon }_{A}\frac{{\alpha }_{A}P({h}_{A}(t))}{1+\alpha P({h}_{A}(t))}{S}_{A}(t)-{m}_{Ab}({h}_{A}(t))-{M}_{A}({h}_{A}(t)){S}_{A1}(t))dt.$$

Here *W*_0_ is the egg carbon weight. The first term in the integrand stands for the energy gain (measured in carbon units) that zooplankton obtain from feeding on phytoplankton with the density *P*. This feeding rate is multiplied by an indicator function ${S}_{A}(t)$, which is equal to 1, when zooplankton actively feed on phytoplankton and is zero otherwise (e.g. during migration or when staying in deep waters). We assume that grazing obeys a Holling type II law with *α*_*A*_ and *α* being coefficients with the well-known ecological meaning^39^. The coefficient *ε*_*A*_ is the food consumption efficiency.

We use the following parametrisation of the vertical distribution of phytoplankton *P* in the water column based on a logistic curve

9 $$P(h)={P}_{0}(\tanh (-\sigma (h-{h}_{p}))+1)/2,$$

where *P*_0_ gives the maximum of the phytoplankton density (we divide *P*_0_ by 2 for normalisation); the parameter *h*_*p*_ is the depth at which *P* is half its maximum. By increasing or decreasing *h*_*p*_ we can explore the influence of food distribution on the strength of vertical migrations. Figure 2E shows that the approximation (9) can be used up to the warm surface waters, i.e. until the depths of 10–15 m. We consider that grazing by zooplankton has only a small impact on the phytoplankton and does not change its profile.

The term ${m}_{Ab}({h}_{A}(t))$ describes losses due to basal metabolism (in carbon units). The metabolic rates for the considered calanoids grazers largely depend on the oxygen concentration which decreases with *h* and it sharply drops as an individual approaches the level of sigma-theta close to 15.7^31^. We use the following parametrisation

$${m}_{Ab}(h)={m}_{A}(\tanh (-{\sigma }_{m}(h-{h}_{m}))+1)/2.$$

Here *h*_*m*_ is a characteristic depth where *m*_*Ab*_ the depth at which the metabolic rate is half its maximum, *σ*_*m*_ describes the sharpness of the transitional layer, and *m*_*A*_ denotes the maximal basal metabolic cost. ${M}_{A}({h}_{A}(t)){S}_{A1}(t)$ denotes the metabolic cost spent on active movements in the water when feeding and moving upwards during vertical migration (ascending phase). The indicator ${S}_{A1}(t)$ is equal to 1 during the feeding and ascending phases of migration, otherwise it is zero. For the descending phase of DVM, we suggest that the organism only spends energy on basal metabolism. The dependence of ${M}_{A}({h}_{A}(t))$ on depth is similar to that of *m*_*Ab*_ with a different maximal value denoted by *M*_*A*0_.

For each stage, the mortality rate within the day is parameterised by

10 $${a}_{i}(v)={\int }_{0}^{1}({\gamma }_{i}(\tanh (-\sigma ({h}_{i}(t)-{h}_{p}))+1)(-\cos \,2\pi t+1)/4+{A}_{i}({h}_{i}(t))+{\gamma }_{i0})dt,$$

where $i=Y,J,A$. Here the first term is the mortality due to visual predators and it varies with the light intensity throughout the day described by $-\cos (2\pi t)+1$. The coefficient *γ*_*i*_ is the product of the constant predator density and its attack rate which is calculated at the highest light intensity (we divide *γ*_*i*_ by 4 for normalisation). Following previous studies, we consider that the density of visual predators is fixed and the risk of predation shows a sigmoidal decrease with depth due to light attenuation^15^. However this assumption is not crucial for the our key findings. The second term stands for extra mortality near the surface and the bottom. It is parameterised as

$${A}_{i}({h}_{i}(t))={\delta }_{u}(\tanh (-{\sigma }_{u}({h}_{i}(t)-{h}_{u}))+1)+{\delta }_{d}(\tanh ({\sigma }_{d}({h}_{i}(t)-{h}_{d}))+1),$$

where *h*_*u,d*_ are the boundaries of the unfavorable zones and the coefficients *σ*_*u,d*_ describe the thickness of the transition layers; *δ*_*u,d*_ characterise of extra mortality when entering an unfavorable zones. Finally, *γ*_*i*0_ is the natural (i.e. non-predation related) mortality.

The growth rates ${r}_{i}(v)$ ($i=Y,J$) are described by the following equation similar to (8)

11 $${r}_{i}(W,v)={\int }_{0}^{1}(\frac{{\varepsilon }_{i}{\alpha }_{i}(W)P({h}_{i}(t))}{1+\alpha P({h}_{i}(t))}{S}_{i}(t)-{m}_{ib}({h}_{A}(t),W)-{M}_{i}({h}_{i}(t),W){S}_{i1}(t))dt,$$

where the model coefficients have similar meaning as in *b*(*v*). Note that unlike the adult stage, where the body weight is assumed to be constant, we include variation of *W* during the developmental stage from the minimal to maximal possible weights. The dependence of *α*_*i*_, *m*_*ib*_ and *M*_*i*_ on *W* is given the allometric expression (12) discussed below; *ε*_*i*_ is the food consumption efficiency for stages $i=Y,J$. The indicator functions *S*_*i*_ and *S*_*i*1_ for stage *i* has the same meaning as those in (8).

The change in the body weight is given by Eq. (4) and maturation times ${\tau }_{i}(v)$ ($i=Y,J$) can be computed by integrating this equation. As such, the maturation time for the young juveniles ${\tau }_{Y}(v)$ will correspond to the minimal carbon weight of juveniles *W*_*J*_, whereas the overall maturation time ${\tau }_{J}(v)$ (i.e. reaching the adult stage) will correspond to the weight of an adult *W*_*A*_.

Note that we also need to formally set the functions ${S}_{i}(t)$ and ${S}_{i1}(t)$. Using the available empirical evidence we can consider that ${S}_{i}(t)=1-\theta (|\dot{x}(t)|-{c}_{0})$, where *θ* is the Heaviside step function, *c*_0_ is a critical vertical velocity of swimming. In other words, vertically moving grazers at a speed higher than *c*_0_ do not consume food. ${S}_{i1}(t)=\theta (\dot{x}(t))$ (here we consider that $\theta (0)=1$); thus this function is zero at the ascending phase and it is switched on at the other phases of DVM.

One can formally search for the exact solution ${h}_{i}(t)$ the optimal control problem. However, it is technically much easier to construct the optimal solution using a piecewise approximation of DVM. Interestingly, this simplification has its important biological rationale since it conforms to empirically observed cases of DVM, indicating that both the descending and the ascending velocities are fairly constant throughout DVM^40,41^. Moreover, our empirical data indicate that zooplankton remain nearly at the same depth when grazing at nighttime and while staying in deep waters. The DVM trajectories can be parameterised as follows $i=Y,J,A$

$${h}_{i}(t)=\{\begin{array}{ll}{H}_{i0} & 0\le t < {t}_{i0},\\ {c}_{i0}(t-{t}_{i0})+{H}_{i0}, & {t}_{i0}\le t < {t}_{i1},\\ {H}_{i1}, & {t}_{i1}\le t < {t}_{i2},\\ -\,{c}_{i1}(t-{t}_{i2})+{H}_{i1}, & {t}_{i2}\le t < {t}_{i3},\\ {H}_{i0}, & {t}_{i3}\le t < t\le 1.\end{array}$$

Here *H*_*i*0_ and *H*_*i*1_ are the shallowest and the deepest depths, respectively; *c*_*i*0_ and *c*_*i*1_ the ascending and descending speeds; times *t*_*ij*_ indicate the end of each phase of movement. By due continuity we have ${t}_{i1}-{t}_{i0}=({H}_{i1}-{H}_{i0})/{c}_{i0}$ and ${t}_{i3}-{t}_{i2}=({H}_{i1}-{H}_{i0})/{c}_{i1}$. For the indicator functions (${c}_{i0} > {c}_{0}$ and ${c}_{i1} > {c}_{0}$) we have ${S}_{i}(t)=1$ with ${t}_{i1}\le t < {t}_{i2}$ and ${S}_{i}(t)=0$ for other *t*; ${S}_{i1}(t)=1$ with ${t}_{i1}\le t < {t}_{i3}$ and ${S}_{i1}(t)=0$ otherwise.

The integral expressions for $b(v),{r}_{i}(v),{a}_{i}(v)$ become algebraic ones and the equations for the optimal parameters can be obtained analytically by differentiation of fitness (7) (see SM4 for detail). One can prove that in the case where ${c}_{i0}={c}_{i1}$, the optimal DVM will be symmetrical with respect to $t=0.5$. This allows us to reduce the number of parameters: each stage is now characterised by only 3 independent parameters.

The parameters for $b(v),{r}_{i}(v),{a}_{i}(v)$ are taken from literature or estimated using our empirical data. They are summarized in Table 1.

**Table 1:** Parameters used in the zooplakton DVM Model along with their units and ranges. For details about origins of parameters see the main text.

Symbol	Meaning	Unit	Range	Default value
*c*_*i*0_ or *c*_*i*1_	Velocity of DVM while ascending or descending, $i=Y,J,A$	m/h	30–50	45
*ε*_*i*_	Food consumption efficiency, $i=Y,J,A$	—	0.6–0.8	0.7
*W*_*A*_	Average carbon weight of adults	*μ*g C	100–150	110
*W*_*J*_	Minimal carbon weight of juveniles	*μ*g g C	20–30	25
*W*_*o*_	Egg carbon weight	*μ*g C	0.2–0.3	0.25
*m*_*A*_	Maximal basal metabolic cost (adults)	*μ*g C ind^−1^ h^−1^	0.04–0.06	0.05
*M*_*A*0_	Maximal overall metabolic costs while actively swimming (adults)	*μ*g C ind^−1^ h^−1^	0.12–0.20	0.13
*h*_*m*_	Depth, where metabolic cost becomes close to basal	m	10–20	12
*α*	Inverse half-saturation density of feeding rate (all stages)	l/*μ*g C	0.005–0.1	0.05
*α*_*A*_	Zooplankton grazing rate (per individual) for adults	l/day	0.05–1.5	1.1
*P*_0_	Maximal phytoplankton density	*μ*g C/l	25–50	30
*σ*	Steepness of decrease of chlorophyll spatial density	1 /m	0.06–0.140	0.125
*h*_*p*_	Half-maximum chlorophyll depth	m	30–50	40
*T*_0_	Reproductive period of adults	day	30–60	40
*γ*_*i*_	Maximal mortality rate due to visual predation, $i=Y,J,A$	1/day	0.4–1.0	0.8
*h*_*d*_	The boundary of the lower unfavourable zone	m	50–150	—
*h*_*u*_	The boundary of the upper unfavourable zone	m	0–30	—
*δ*_*d*_	Increase in mortality in the lower unfavourable zone	1/day	—	2
*δ*_*u*_	Increase in mortality in the upper unfavourable zone	1/day	—	0.5
*σ*_*u,d*_	Steepness of decrease of mortality while entering unfavourable zones	1 /m	0.05–0.4	0.2
*σ*_*m,M*_	Steepness of decrease of metabolic rates with depth	1 /m	0.04–0.065	0.65

The velocity of migration *c*_*i*0_ and *c*_*i*1_ is estimated as 30–50 *m*/*h* for all stages using acoustic scattering across the water column^40,41^. From the existing data, we can assume that the magnitudes of upwards and downwards migration velocities are close to each other.

The maximal mortality rate *γ*_*i*_ due to visual predation during day is highly variable since it is a product between the predator attack rate and the predator density. In this paper, we consider it to vary as 0.6–1.0 $1/day$^14,15^. The natural mortality rate is assumed to be high for early stages (CI-CIII) (${\gamma }_{Y0}=0.11/day$) and negligibly small for later stages (CIV-CVI), ${\gamma }_{i0}\approx 0$. The thickness of transition layers *σ*_*u,d*_ in the profiles of the temperature and the sigma-theta curve can be estimated from our data which gives, respectively, *σ*_*u*_ = 0.05–0.21/*m* and *σ*_*d*_ = 0.05–0.41/*m* (see SM3 for detail). The maximal values of mortality rates (given by 2*δ*_*d,u*_) are hard to assess. For example, in the absence of oxygen, a copepod individual dies within less than 1 hour, which makes *δ*_*d*_ extremely high. Here, for the sake of efficiency of numerical procedure, we consider some smaller values as ${\delta }_{d}=21/day$: increasing *δ*_*d*_ does not affect the modelling results since the optimal trajectory of DVM does not enter the dangerous hypoxic zone. We also set ${\delta }_{u}=0.51/day$ for the upper unfavorable zone with high temperature. This value is within the reported ranges^42^. Using our observation, we explore the following ranges *h*_*d*_ = 50–150 *m* and *h*_*u*_ = 0–30 *m*.

The parameters describing phytoplankton distribution (9) in the water column are estimated from our data averaged across all years (see Fig. 2E). This gives ${P}_{0}=0.6\pm 0.2\,\,mgChla/{m}^{3}$ which roughly corresponds to 15–30 $\mu g\,C/l$, ${h}_{p}=40\pm 8\,m$ and $\sigma =0.1\pm 0.04\,1/m$. The assimilation efficiency *ε*_*i*_ varies from 0.6–0.8; for simplicity, we consider it to be the same for all developmental stages^43^. The coefficients *α*_i_ and *α* describing the functional response of grazers can be estimated as $0.02 < {\alpha }_{i} < 0.3\,\,l/\mu gC/day$ and $0.005 < \alpha  < 0.05$ $\mu gC/l$^44^. To describe consumption rate per individual, we need to multiply *α*_*i*_ by the average carbon weight of an individual at a given stage. For *C. euxinus* we suggest that the carbon weight of an adult female (stage CVI) is approximately *W*_*A*_ = 100–150 *μgC*^32^; this allows us to consider the following range of *α*_*A*_ = 1.0–2.1 *l*/*day*. The values of *α*_*i*_, *m*_*i*_ and *M*_*i*0_ for other stages are calculated using the following allometric scaling law^45^

12 $${\alpha }_{i}(W)={C}_{1}{W}^{0.8},{m}_{i}(W)={C}_{2}{W}^{0.8},{M}_{i0}(W)={C}_{3}{W}^{0.8},$$

where *W* the carbon weight of an individual, *C*_*j*_ are constant usually depending on the temperature. For *C. euxinus* we consider that the minimal carbon weight of the older juveniles (stage CIV) is *W*_*J*_ = 20–30 *μgC*, whereas for the young juveniles (stages CI-III) the minimal weight is approximately equal to ${W}_{Y}=1.2\,\,\mu gC$^23,32^. Here we do not explicitly model the nauplii stages resulting in growth from an egg to the start of stage CI.

The maximal basal metabolic cost and the maximal overall metabolic costs for actively swimming adults can be estimated, respectively as *m*_*A*_ = 0.05–0.07 *μgC* *ind*^−1^ *h*^−1^ and *M*_*A*0_ = 0.12–0.20 *μgC* *ind*^−1^ *h*^−1^ ^31^, see also SM3. The metabolic costs *m*_*i*_ and *M*_*i*0_ for the other developmental stages were calculated using (12). The characteristic depth *h*_*m*_, where the overall metabolic cost becomes close to basal cost, is assumed to be ${h}_{m}={h}_{d}-\delta h$, where *δh* = 15–20 *m* (see SM3); the thickness *σ*_*m*_ of the layers where metabolic costs approach zero can be estimated from data of this study (see SM3) and other works^31^, which gives ${\sigma }_{m,M}=0.04-0.065\,1/m$. The maximal possible reproductive period of adults can be estimated as *T*_0_ = 30–60 *days*^46^.

To model DVM of the other zooplankton species *P. elongatus*, we used the parameters as in Table 1 and apply the same allometric relation for the following body and egg weights: ${W}_{Y}=0.25\,\mu gC$, ${W}_{J}=0.8\,\mu gC$ and ${W}_{A}=3.0\,\mu gC$ and the egg weight ${W}_{0}=0.12\,\mu gC$^47^. The maximal possible reproductive period of *P. elongatus* was estimated as *T*_0_ = 30–60 *days*.

#### Optimal trajectories of DVM

Numerical optimization of fitness *J* was done using the MATLAB function ‘fminsearch’ which implements the Nelder-Mead simplex algorithm of optimisation. The eigenvalues in (7) were computed numerically using the MATLAB function ‘fzero’ (we also found that the dominant eigenvalue is always real). To make sure that we find the global maximum, we consider different starting points for optimisation. We also checked the correctness of the optimisation results by numerically solving the equations expressing the first derivatives of fitness which should vanish at a point of maximum (see SM4 for detail).

**Figure 3:**
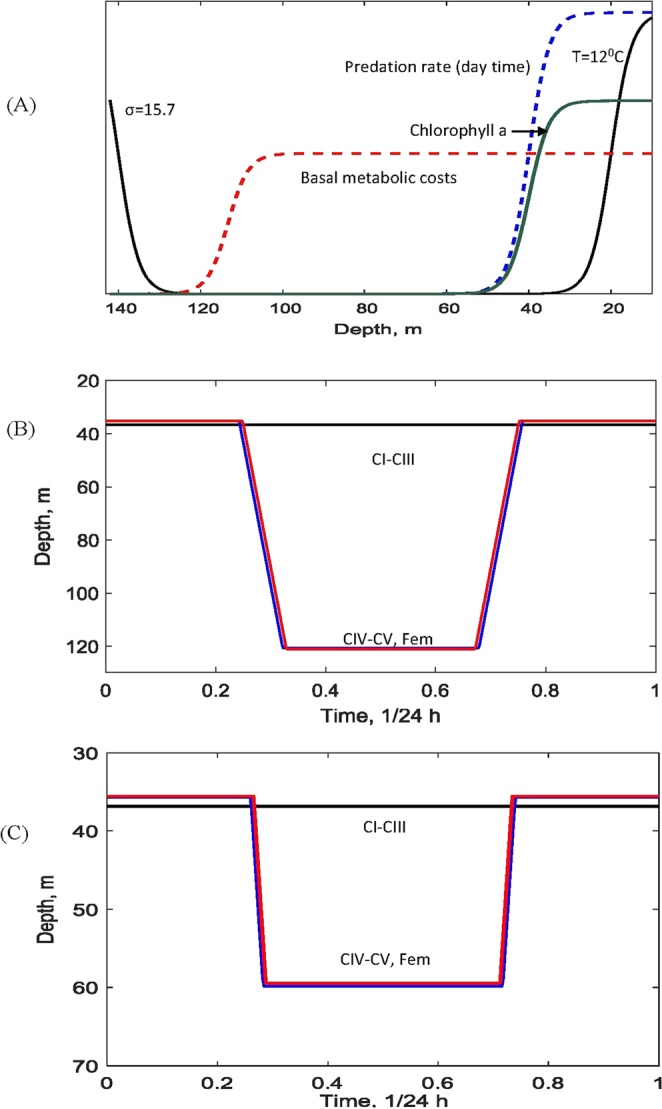
(**A**) The abiotic and biotic components across the water column which constitute the environment for zooplankton grazers in summer (${h}_{d}=140\,$m, ${h}_{u}=20\,$m). The scaling in the vertical direction for each curve can be obtained considering the maximal values provided in Table 1. (**B**) Typical pattern of optimal DVM in the model for *C. euxinus* in the environment shown in panel (**A**). The other parameters are given in Table 1. (**C**) Optimal DVM of *C. euxinus* constructed for the hypothetical modelling scenario with a depth-independent basal metabolic cost. In (**B**,**C**) blue and red lines denote, respectively, females (stage IV) and juveniles (CVI-CV).

Figure 3A represents the model environment which zooplankton grazers would potentially experience in a typical summer (with ${h}_{d}=140\,m$ and ${h}_{u}=20\,m$): this includes profiles of their food, chlorophyll a, their daytime predation risk, the temperature and sigma-theta curves as well as variation of basal metabolic costs. Patterns of optimal DVM for the environment in Fig. 3A are shown in Fig. 3B calculated for *C. euxinus*. The graphs of DVM for *P. elongatus* are rather similar and we do not show them for brevity. One can see that early stages (CI-CIII, *i* = *Y*) do not migrate and stay in shallow waters throughout the day (${H}_{Y1}={H}_{Y0}\approx 35\,m$). Unlike previous explanation in the literature, which suggested that zooplankton of early stages do not have enough energy to perform vertical migration, we found that they do not undergo DVM due to a high predator-independent mortality for the given stages. Our model predicts that it becomes more optimal for very early developmental stages to stay and feed in shallow waters to maximise their growth rate to be able to reach the next stages as soon as possible: stages CIV-VI generally have a lower natural mortality rate. On the contrary, the older developmental stages (CIV-VI, $i=J,A$) perform night feeding in upper layers and stay in deeper waters during the day time. The obtained pattern is close to the one observed empirically (cf. Fig. 1A,B). Interestingly, stages CIV-CV and adult females (CVI) show very close DVM trajectories both in terms of migration times and migration depths. The DVM predicted by the model are triggered by daily variation of the predation load: they disappear when zooplankton mortality rate becomes constant. The choice of the optimal depths of migration is a more complicated issue and is addressed in detail below.

Our simulation shows that the maximal depth of optimal DVM for the older stages is determined by the spatial gradient of metabolic costs rather than predation. Indeed, the depths at which zooplankton stay during the daytime are characterised by low basal metabolic rates due to low oxygen concentration ($h\approx 120\,m$), (cf. Fig. 3A). On the other hand, the consumption rate of zooplankton by visual predators becomes extremely small starting at much shallower depths ($h\approx 55\,m$) due to light attenuation and has no further effect on the maximal DVM depth. To explore the relative roles of predation and metabolism on DVM, we completed simulations using the same model parameters but assuming a depth-independent metabolic cost. We found that DVM is still observed in this case, but the migration depths during the day time become shallower. The DVM for ${m}_{ib}={m}_{i}=const$ ($i=Y,J,A$) is shown in Fig. 3C demonstrating much shallower depths of migration as compared to depth-dependent metabolic cost in Fig. 3A.

**Figure 4:**
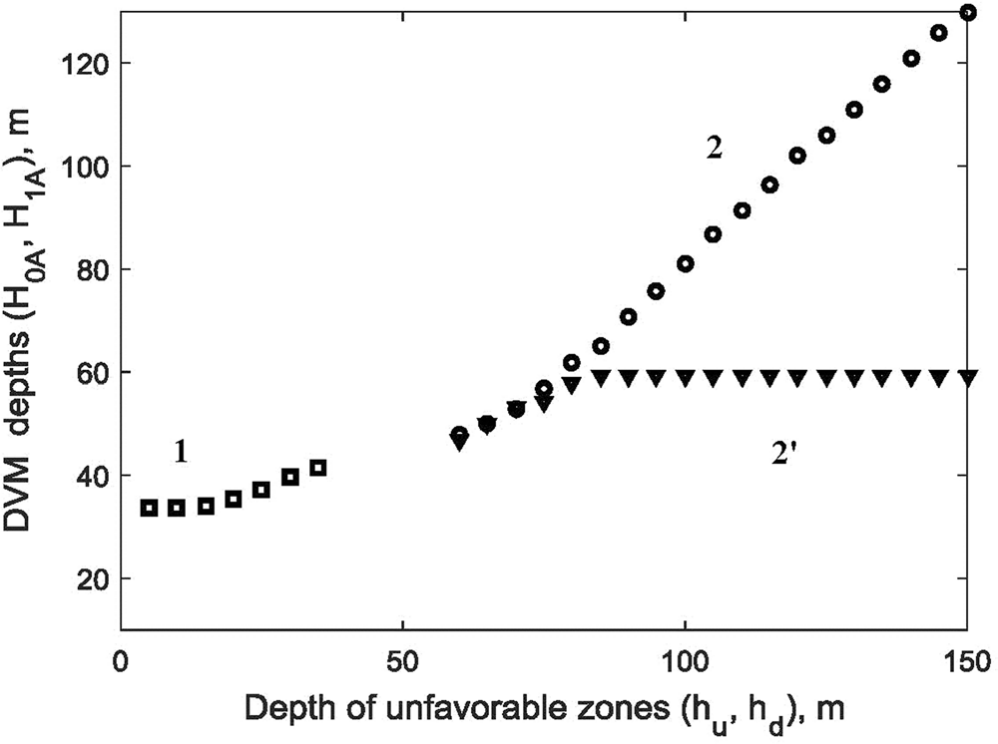
Connection between the upper and lower unfavorable zones (*h*_*d*_ and *h*_*u*_) in the water column and the upper and the lower depths (${H}_{A0},{H}_{A1}$) of females in the model obtained for *C. euxinus*. The impact of variation of *h*_*u*_ (for a constant ${h}_{d}=140\,$m) on *H*_*A*0_ is shown via empty squares (curve 1). The impact of *h*_*d*_ (${h}_{u}=20\,$m) on *H*_*A*1_ is denoted by circles 2 (constructed for spatially variable metabolic costs) or by triangles 2′ (constructed for spatially constant metabolic costs). The other parameters are taken from Table 1.

We further investigated the influence of the locations of the upper and lower unfavorable zones characterised by *h*_*d*_ and *h*_*u*_ on the depths of DVM denoted by ${H}_{A0},{H}_{A1}$. The results are presented in Fig. 4. In this figure, the dependence of *h*_*u*_ on *H*_*A*0_ is denoted by squares 1, whereas the influence of *h*_*d*_ on *H*_*A*1_ is denoted either by circles 2 (constructed for spatially variable metabolic costs) or by triangles 2′ (constructed for a hypothetic regime with spatially constant metabolic costs). As one can see, for spatially variable metabolic costs, an increase of *h*_*d*_ starting from ($h\approx 60\,m$) will result in a pronounced increase of the migration depth. However, such an increase does not occur for the scenario with spatially constant metabolic costs. On the other hand, when the depth *h*_*u*_ of the unfavorable zone approaches the surface, this only has a partial effect on the upper depth *H*_*A*0_ of DVM. This is explained by the fact that a high trophic pressure by visual predators near the surface does not allow grazers to feed in shallow waters even if the temperature regime becomes comfortable. Interestingly, these model predictions correlate well with our empirical observation shown in Fig. 2B,C.

**Figure 5:**
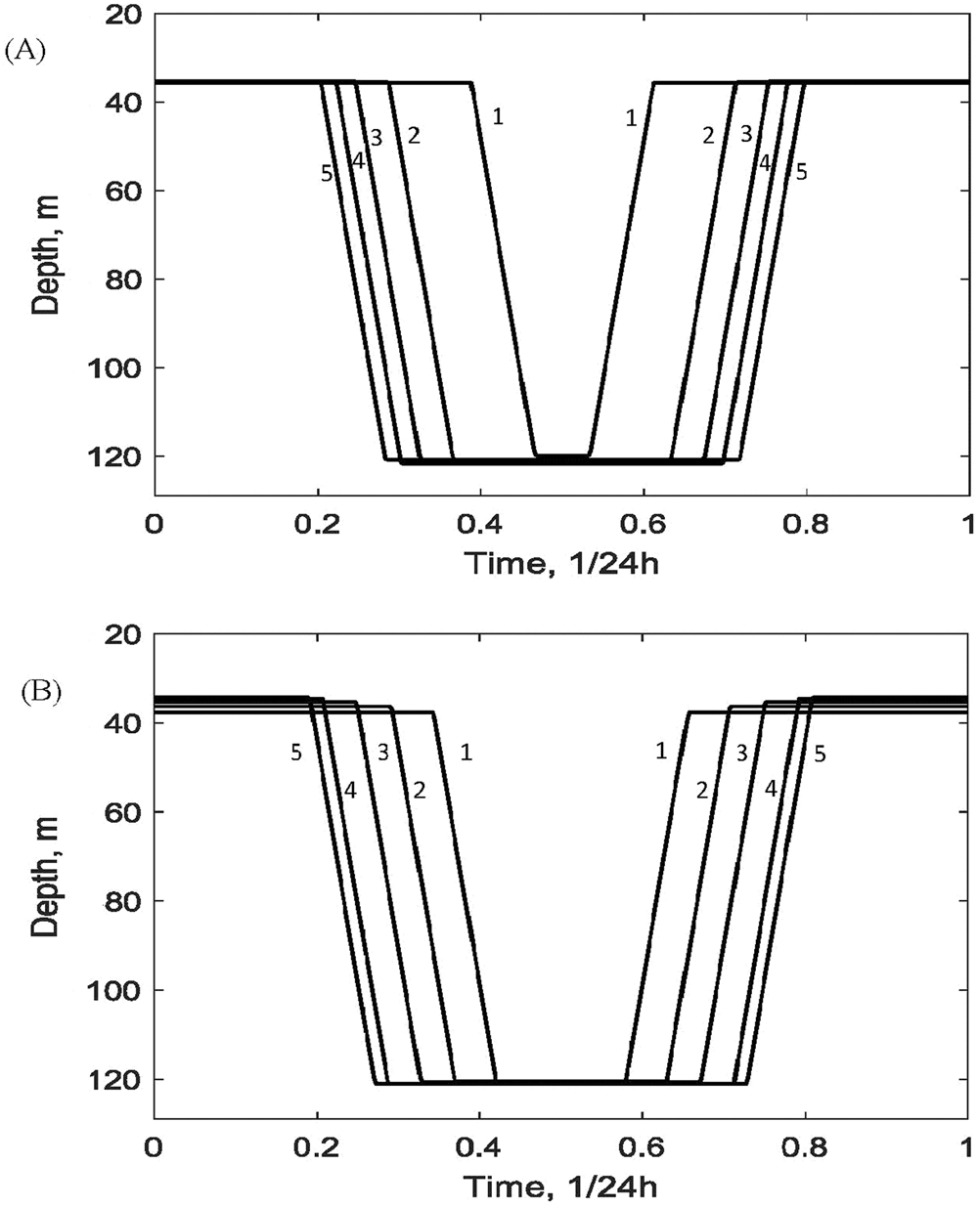
(**A**) Influence of predation pressure on the optimal DVM of females (CVI) of *C. euxinus* in the model. Trajectories (1–5) correspond to ${\gamma }_{A}=0.44;0.60;0.80;1.0;1.2$ 1/day, respectively. (**B**) Influence of the available food on the optimal DVM of females (CVI) of *C. euxinus* in the model. Trajectories (1–5) correspond to ${P}_{0}=36;30;25;20;16$ *μ*g C/l, respectively. Here ${h}_{d}=140\,$m, ${h}_{u}=20\,$m, the other parameters are as in Table 1.

We also investigated the dependence of DVM on other key model parameters. Varying the predation pressure on zooplankton, we found that an increase in *γ*_*i*_ results in larger time spent by grazers in deep waters (Fig. 5A) without significantly affecting the depths of migration. At low predator pressure (e.g. ${\gamma }_{i}=0.1\,1/day$) migration ceases completely. An increase in the available food as described by *P*_0_ reduces the time spent by grazers in deep waters which is shown in Fig. 5B. The model predicts that migration should cease at high phytoplankton density (${P}_{0} > 45\,\mu g\,C/l$). The model predicts that variation of the reproductive period *T*_0_ within the realistic parameter range does not largely affect DVM.

Finally, we found that for both considered zooplankton species their daily food consumption and their maturation times *τ*_*i*_ correspond well to estimates from the literature^31–33,48^. This provides extra credits to support our model findings.

## Discussion

Fitness has always been a fundamental concept both in empirical and theoretical evolutionary biology following the seminal idea of Sewall Wright about a hypothetical adaptive fitness landscape^49^. The metaphor of climbing uphill in an evolutionary landscape to reach a local peak is so popular in the literature because it is compelling and easily graspable. However, the initial idea has been criticized because the shape of the fitness landscape in real biological systems is often non-stationary and constantly varies in the course of evolution due to a permanent dynamical feedback between individuals and their environment^50^. Here we revise the concept of evolutionary fitness using the original idea of S. Wright but allowing for dynamical feedbacks. As a result, the theory still predicts that long-term selection for a best strategy or strategies should maximise some function or functional which we understand as the fitness. The revised definition of fitness takes into account dynamical feedbacks from individuals of the same population and the environment consisting of other species and abiotic factors, and is based on introducing a ranking order of strategies, defined as the long-term ratio of generalised densities (1) of some measure in an arbitrary function space. Such densities may not have the same meaning as population densities, because they can generally be functions of the ‘true’ population densities (cf. SM1(ii)). We argue that the mathematical formalism proposed here is generic and can be equally applied to modelling the processes of selection and evolution in chemistry, turbulence theory, chemical kinetics, cultural evolution or economics.

The population fitness *J* introduced in this paper allows us to formulate the variational principle of evolutionary modelling: the strategies remaining in the system after long-term selection should correspond to the maximum of the fitness functional across the strategies initially present. An example of derivation of the variational equations providing optimal parameters is shown for the DVM model in SM4. Note that the framework allows us to consider both scalar- and function-valued strategies *v*: in the latter case, the variational equations will be optimal control theory equations. Establishing variational principles in modelling biological evolution has a long history with various approaches proposed^6,8,51,52^. For example, optimisation principles in evolution have been earlier suggested in the adaptive dynamics framework, where it was found that evolution will optimise the invasion fitness (the per-capita rate of growth of a rare mutant introduced into a resident population) in the case where the environment affects fitness in an effectively monotone one-dimensional manner^52^. However, generic classes of models where this property holds are still poorly understood^53^. The concept of evolutionary optimization used here is different from that used in the adaptive dynamics framework; in particular, it does not consider an invasion-replacement paradigm^52,53^. Overall, unlike the situation in mechanics, thermodynamics or optics, a unified variational principle for modelling selection in biological systems is still in its mathematical infancy and we believe that this paper makes a further step in this direction.

The proposed theoretical approach allows us to more easily define evolutionary fitness for population models with structuring (including systems with delay) which can be a challenge in other modelling frameworks. As an important practical example, we derived the expression for evolutionary fitness *J* for the population model of von Foerster type with age- or size-structuring with an arbitrary mortality term. This type of equation is widely used in the modelling literature and possible applications of the theory can go well beyond the considered example of zooplankton DVM. The derived fitness may include as many developmental stages as possible (see SM1(ii)), or we can refine the behavior or life-history within a given developmental stage by introducing sub-stages. Using a similar approach one can obtain evolutionary fitness for a structured population model with *n* stages under some other modelling settings: for example, by considering a system of ODEs in which each stage is described via a separate differential equation^16^.

We apply our theoretical findings to modelling optimal diel vertical migration (DVM) of zooplankton. DVM is the largest synchronised animal movement on Earth and has tremendous consequences for marine dynamics, fisheries and the ocean carbon pump^19,20^. The need for creating new models of DVM is dictated by the fact that in previous models, patterns of optimal behaviour of zooplankton were obtained by a direct maximization of some criterion such as venturous revenue, reproductive value or predation pressure^7–10,12,14,15^, and in each case the choice of criterion was based on conventional wisdom or the personal preference of the researcher. Here are we not claiming, however, that all previous models of DVM are necessarily wrong, but trying to point out the main problem of setting some initially prescribed common sense criterion of optimality. Such a criterion often does not demonstrate a straightforward connection between the maximization or minimization of some factor and the long-term population growth, especially in the presence of various nonlinear feedbacks and trade-offs. On the contrary, according to the introduced definition of fitness, the resultant patterns of optimal DVM would be a product of long-term selection of the subpopulation employing the given strategy. As such the optimal DVM is only determined by the underlying population model and the parameterisation of the model coefficients.

The DVM model was validated using long-term observation of the migration of herbivorous zooplankton in the north-eastern Black Sea (2007–2014). The model may provide solutions to several long-term open questions about the nature of DVM in this ecosystem, which is characterized by unique hydrological and biochemical regimes, in particular by the existence of a permanent anoxia zone and the low depth of light penetration^54^. Firstly, our model confirms that the intense variation of visual predation due to the periodic change in light intensity is the main trigger of DVM, a fact which was reported in earlier DVM models. Interestingly, however, the predation pressure should be supercritical: sufficiently low pressure results in cessation of DVM. Secondly, our model predicts that predation does not entirely determine the amplitude of DVM. Namely, we find that other components of the environment such as oxygen concentration and the temperature may play a crucial role, almost tripling the amplitude of DVM compared to the case with depth-independent metabolic costs (cf. Fig. 3B,C).

The main model-based explanation of unexpectedly large amplitudes DVM in the Black Sea in summer is that staying in deep waters with low metabolic costs is beneficial for grazers since this allow them to substantially reduce their energy losses (see Fig. 3A). As a result, the deepest migration depth throughout the day can be as high as 120–130 m whereas the light intensity is reduced up to 1–2% already at depths of 50–60 m^54^. The model therefore provides a theoretical basis to explain the large amplitude DVM observed in the Black Sea, which was previously a matter of discussion in the literature. Note that the key role of metabolic costs on the amplitude of DMV has previously been suggested by direct laboratory measurements of the metabolic rates of grazers under different oxygen conditions^31^. We argue, however, that the conclusion made based on the laboratory studies^31^ that staying in deep waters with low basal metabolic costs during the daytime should always be beneficial for grazers is not quite as straightforward as it seems. The model-based computation shows that fitness is maximized by staying at the low oxygen boundary only if (i) the cost of migration is not high and (ii) the drop in metabolic costs at the edge of the anoxic zone should be supercritical, otherwise large amplitude DVM becomes counterproductive. On the other hand, predation rate, natural mortality and food availability are crucial factors affecting the amplitude of DVM (see Fig. 5). These conclusions could not be made solely through empirical observation and laboratory experiments.

Another counter-intuitive conclusion from the DVM model concerns the reason that early copepodite stages (CI-CIII) do not migrate. In some previous studies^7^, this absence of migration was postulated, and has been confirmed in a large number of study cases^23^. A widespread opinion in the literature is that the main reason for the absence of DVM in early stages is the lack of sufficiently large amounts of energy-per-biomass in stages CI-CIII to be able to perform DVM. However, in our model this behaviour is an emergent property (see also Fig. 1), and we found that it is the high mortality rates rather than a lack of energy that keeps the earlier stages in shallow waters. For example, we found that reducing the natural mortality in the model results in emergence of DVM for CI-CIII even if we increase the costs of migration (we do not show this result for brevity).

Finally, we would like to give a general warning about the interpretation of the influence of several factors on DVM predicted by the model, for example, the alteration and even disappearance of DVM when the food supply in shallow waters is high enough or when predation is sufficiently low (Fig. 5). We argue that we should interpret these theoretical predictions as the optimal responses of grazers to some long-term trends rather than to fast change in the environmental conditions. Our data show that the vertical profile of chlorophyll in the considered ecosystem is highly variable from year to year, even within a single season (the data are not shown), and the same concerns the predation pressure by visual predators. A number of studies of empirical observation of DVM in the given ecosystem, however, demonstrate a constant pattern of regular migration across both days and seasons. This supports the idea that the DVM pattern of grazers observed in the model is only optimal on average and can be suboptimal on a daily basis. On the other hand, one should also consider the presence of other species dynamically. Including dynamical predators, for example, with abundance depending on that of zooplankton herbivores, may result in DVM being beneficial in ecosystems with abundant food for zooplankton^16^. In the current model settings with a single population model, this can be modeled via a trade-off between predation rates and food density, for example.

Among future perspectives for modelling selection processes based on the proposed framework we can cite the following. It would be straightforward, wherever possible, to establish classes of models where the existence of evolutionary fitness can be demonstrated. We predict that some complications may occur in the case where fitness depends on initial conditions and where the ranking order can not be properly defined. Moreover, in the case where it is not possible to analytically derive the expression for evolutionary fitness, such as in multi-species models, proper computational methods should be developed. Finally, regarding the particular ecological study case of DVM, it would be interesting to extend the considered model of zooplankton migrations to some more realistic settings by explicitly describing each of the 6 developmental stages as well as including variation in physiological conditions in each stage such as lipid reserves, moulting and starvation, as was done in some previous studies^7^. We are planning to address the above issues in our future works.

## Supplementary information

Supplementary Material

## Data Availability

All data generated or analysed during this study are included in this published article (and its Supplementary Material).
