# Supplementary material for: Modelling optimal behavioural strategies in structured populations using a novel theoretical framework
Source: Sci Rep. 2019 Oct 21;9:15020. doi: 10.1038/s41598-019-51310-w (PMC6803682; doi:10.1038/s41598-019-51310-w)
Supplement: Supplementary file 1 — Supplementary Material [file 41598_2019_51310_MOESM1_ESM.docx]

**Supplementary Materials (SM) for the manuscript ‘Modelling optimal behavioral strategies in structured populations using a novel theoretical framework’**

Andrew Yu. Morozov, Oleg A. Kuzenkov and Elena G. Arashkevich

In this supplementary material we provide details of the rigorous mathematical formulation of evolutionary fitness, in particular this includes: proofs of Theorems 2,4 (**SM1**); technical comments on empirical observation of diel vertical migration (DVM) of zooplankton in the Northern-Eastern Black Sea (**SM2**); empirical data on vertical profiles for some abiotic factors which potentially have a large impact on zooplankton DVM in the considered ecosystem (**SM3**); derivation of the equations for the optimal parameters of DVM (**SM4**).

**Short table of Contents**

Proofs of Theorems 2 and 4 on formalisation of fitness (SM1)……...............…….1

Empirical observation of DVM of zooplankton in the Black Sea (SM2) .…….…..5

Empirical data on abiotic factors affecting DVM (SM3)…………..………………9

Derivation of equations for optimal parameters of DVM (SM4)…….……………12

**SM1**

1. Proof of Theorem 2.

We assume that $v'\succ w'$. In accordance to Definition 1, there should be neighbourhoods *O*(*v'*) and *O*(*w'*) of points *v'* and *w'* such that the ratio of the densities *η*(*w*,*t*)/ *η*(*v*,*t*) tends to zero uniformly in *O*(*v'*) and *O*(*w'*). In other words, for any *ε* >0 there should be a time *T* >0 such that for any *w* from *O*(*w'*) and for any *v* from  *O*(*v'*) for any *t* >*T* the following inequality holds

$\frac{\eta(w,t)}{\eta(v,t)}<$ *ε.*

Moreover, the measure *µ^*^* of any open measurable set (except the empty set Ø) is larger than 0. Therefore, $\mu^{*}\left( O\left( v^{'} \right) \right)=\int_{O\left( v^{'} \right)} \mu^{*}dv=\beta>0$and *η(w,t)<εη(v,t)* .

By integrating the last inequality for the densities in the neighbourhood *O*(*v'*) we obtain

$$\int_{O\left( v^{'} \right)} \eta(w,t)\mu^{*}dv=\beta\eta\left( w,t \right)\leq\varepsilon\int_{O\left( v^{'} \right)} \eta\left( v,t \right)\mu^{*}dv\leq\varepsilon\int_{V} \eta\left( v,t \right)\mu^{*}dv.$$

Since $\int_{V} \eta\left( v,t \right)\mu^{*}dv<c$ we have

$$\eta\left( w,t \right)<\frac{c\varepsilon}{\beta} .$$

Thus the density *η(w,t)* in the neighbourhood *O*(*w'*) of *w'* uniformly tends to zero for large times. This finishes the proof of the Theorem.

1. Proof of Theorem 4.

We introduce the integral quantities expressing the total population densities for stage *i*.

$S_{i}\left( v,t \right)=\int_{\tau_{i}}^{\tau_{i+1}} \rho\left( v,\tau,t \right)d\tau$, $0\leq i\leq n+1$, $\tau_{0}=0.$

Then we rewrite equation (5) from the main text in terms of $S_{i}\left( v,t \right)$:

$$\frac{dS_{0}(v,t)}{dt}+\varrho\left( v,\tau_{1},t \right)-\varrho\left( v,0,t \right)=-a_{0}\left( v \right)S_{0}(v,t)-R\left( v \right)y(t)S_{0}(v,t)$$

$$\frac{dS_{1}(v,t)}{dt}+\varrho\left( v,\tau_{2},t \right)-\varrho\left( v,\tau_{1},t \right)=-a_{1}\left( v \right)S_{1}(v,t)-R\left( v \right)y(t)S_{1}(v,t)$$

………

$$\frac{dS_{n}(v,t)}{dt}+\varrho\left( v,\tau_{n+1},t \right)-\varrho\left( v,\tau_{n},t \right)=-a_{n}\left( v \right)S_{n}(v,t)-R\left( v \right)y(t)S_{n}(v,t)$$

For the densities $\varrho\left( v,\tau,t \right)$ at age $\tau$ we have

$$\varrho\left( v,\tau,t \right)=\varrho\left( v,0,t-\tau\right)exp\left( -a_{0}\left( v \right)\tau-R(v)\int_{t-\tau}^{t} y\left( t \right)dt \right), 0\leq\tau\leq\tau_{1}$$

$$\varrho\left( v,\tau,t \right)=\varrho\left( v,\tau_{1},t-(\tau-\tau_{1}) \right)exp\left( -a_{1}\left( v \right)(\tau-\tau_{1})-R(v)\int_{t-(\tau-\tau_{1})}^{t} y\left( t \right)dt \right), \tau_{1}\leq\tau\leq\tau_{2}$$

…….

$\varrho\left( v,\tau,t \right)=\varrho\left( v,\tau_{n},t-(\tau-\tau_{n}) \right)exp\left( -a_{n}\left( v \right)(\tau-\tau_{n})-R(v)\int_{t-(\tau-\tau_{n})}^{t} y\left( t \right)dt \right), \tau_{n}\leq\tau\leq\tau_{n+1}$.

The boundary condition is now given by

$\varrho\left( v,0,t \right)=\sum_{i=1}^{n} b_{i}(v)S_{i}(v,t)$.

Next we substitute $\varrho\left( v,\tau,t \right)$ into the equations for *S_i_* to obtain

$$\frac{dS_{0}(v,t)}{dt}=\sum_{i=1}^{n} b_{i}\left( v \right)S_{i}\left( v,t \right)-\sum_{i=1}^{n} b_{i}\left( v \right)S_{i}\left( v,t-\tau_{1} \right) exp\left( -a_{0}\left( v \right)\tau_{1}-R\left( v \right)\int_{t-\tau_{1}}^{t} y\left( t \right)dt \right)-a_{0}\left( v \right)S_{0}(v,t)-R\left( v \right)y(t)S_{0}(v,t)$$

$$\frac{dS_{1}\left( v,t \right)}{dt}=\sum_{i=1}^{n} b_{i}\left( v \right)S_{i}\left( v,t-\tau_{1} \right)exp\left( -a_{0}\left( v \right)\tau_{1}-R\left( v \right)\int_{t-\tau_{1}}^{t} y\left( t \right)dt \right)$$

$$-{\sum_{i=1}^{n} b_{i}\left( v \right)S_{i}\left( v,t-\tau_{2} \right)exp\left( -a_{0}\left( v \right)\tau_{1}-a_{1}\left( v \right)(\tau_{2}-\tau_{1})-R\left( v \right)\int_{t-\tau_{2}}^{t} y\left( t \right)dt \right)-a}_{1}\left( v \right)S_{1}(v,t)-R\left( v \right)y(t)S_{1}(v,t)$$

……

$$\frac{dS_{n}\left( v,t \right)}{dt}=\sum_{i=1}^{n} b_{i}\left( v \right)S_{i}\left( v,t \right) S_{i}\left( v,t-\tau_{n} \right)exp\left( -\sum_{k=0}^{n-1} a_{k}\left( v \right)\left( \tau_{k+1}-\tau_{k} \right)-R\left( v \right)\int_{t-\tau_{n}}^{t} y\left( t \right)dt \right)$$

$$-{\sum_{i=1}^{n} b_{i}\left( v \right)S_{i}\left( v,t \right) S_{i}\left( v,t-\tau_{n} \right)exp\left( -\sum_{k=0}^{n} a_{k}\left( v \right)\left( \tau_{k+1}-\tau_{k} \right)-R\left( v \right)\int_{t-\tau_{n+1}}^{t} y\left( t \right)dt \right)-a}_{n}\left( v \right)S_{n}\left( v,t \right)-R\left( v \right)y\left( t \right)S_{n}\left( v,t \right).$$

We introduce new variables which we interpret as the generalised densities

$F_{i}(v,t)$=$S_{i}\left( v,t \right)exp\left( R(v)\int_{0}^{t} y\left( t \right)dt \right),$ $0\leq i\leq n$.

In new variables, the model equations become

$$\frac{{dF}_{0}(v,t)}{dt}=\sum_{i=1}^{n} b_{i}\left( v \right)F_{i}\left( v,t \right)-\sum_{i=1}^{n} b_{i}\left( v \right)F_{i}\left( v,t-\tau_{1} \right) exp\left( -a_{0}\left( v \right)\tau_{1} \right)-a_{0}\left( v \right)F_{0}\left( v,t \right),$$

$$\frac{{dF}_{1}(v,t)}{dt}=\sum_{i=1}^{n} b_{i}\left( v \right)F_{i}\left( v,t-\tau_{1} \right) exp\left( -a_{0}\left( v \right)\tau_{1} \right)-\sum_{i=1}^{n} b_{i}\left( v \right)F_{i}\left( v,t-\tau_{2} \right) exp\left( -a_{0}\left( v \right)\tau_{1}-a_{1}\left( v \right)(\tau_{2}-\tau_{1}) \right)-a_{1}\left( v \right)F_{1}\left( v,t \right),$$

……

$$\frac{{dF}_{n}(v,t)}{dt}=\sum_{i=1}^{n} b_{i}\left( v \right)F_{i}\left( v,t-\tau_{n} \right) exp\left( -\sum_{k=0}^{n-1} a_{k}\left( v \right)\left( \tau_{k+1}-\tau_{k} \right) \right)-\sum_{i=1}^{n} b_{i}\left( v \right)F_{i}\left( v,t-\tau_{2} \right) exp\left( -\sum_{k=0}^{n} a_{k}\left( v \right)\left( \tau_{k+1}-\tau_{k} \right) \right)-a_{n}\left( v \right)F_{n}\left( v,t \right).$$

We now seek the solution of the above system as a sum of $e_{i}exp\left( \lambda_{i}t \right)$, where $e_{i}=\left( e_{i0},e_{i1},\ldots,e_{in} \right)$ is a constant vector. The characteristic equation for the eigenvalues $\lambda$ becomes the following transcendental equation

$$\det\left(\begin{matrix} -a_{0}-\lambda& b_{1}\left(1-\exp\left(f_{1}\right)\right) & ... & b_{n}\left(1-\exp\left(f_{1}\right)\right) \\ 0 & b_{1}\left(\exp\left(f_{1}\right)-\exp\left(f_{2}\right)\right)-a_{1}-\lambda& ... & b_{n}\left(\exp\left(f_{1}\right)-\exp\left(f_{2}\right)\right) \\ ... & ... & ... & ... \\ 0 & b_{1}\left(\exp\left(f_{n}\right)-\exp\left(f_{n+1}\right)\right) & ... & b_{n}\left(\exp\left(f_{n}\right)-\exp\left(f_{n+1}\right)\right)-a_{n}-\lambda\end{matrix}\right)=0$$

where$f_{i}=-\tau_{i}\lambda-\sum_{k=0}^{i-1} a_{k}\left( v \right)\left( \tau_{k+1}-\tau_{k} \right)$, $0\leq i\leq n$ .

The general solution of the model in terms of *S_i_* is given by an infinite series

$$\left( F_{0},F_{1},\ldots,F_{n} \right)=\sum_{i=1}^{\infty} c_{i}\exp\left( \lambda_{i}t \right)e_{i}.$$

We then introduce the following global variables: *S*($v,t$)=*S_0_*($v,t$)+*S_1_*($v,t$)+...+*S_n_*($v,t$) and *F*($v,t$)=*F_0_*($v,t$)+*F_1_*($v,t$)+...+*F_n_*($v,t$). To characterise the presence of the strategy $v$ we introduce the generalised density $\eta\left( v,t \right)= S^{\frac{1}{R\left( v \right)}}(v,t)$ which satisfies the assumptions stated in Section 2.1. In this case, comparison of the strategy $v$ with another strategy *w* will be done via comparison of$\eta\left( v,t \right)$

$$\lim_{t\to\infty} \frac{\eta(v,t)}{\eta(w,t)}=\lim_{t\to\infty} \frac{S^{\frac{1}{R\left( w \right)}}(w,t)}{S^{\frac{1}{R\left( v \right)}}(v,t)}=\lim_{t\to\infty} \frac{F^{\frac{1}{R\left( w \right)}}(w,t)}{F^{\frac{1}{R\left( v \right)}}(v,t)}$$

$$=\lim_{t\to\infty} \frac{\left( \sum_{i=1}^{\infty} c_{i}(w)exp(\lambda_{i}(w)t)\sum_{j=0}^{n} e_{ij}(w) \right)^{1/R(w)}}{\left( \sum_{i=1}^{\infty} c_{i}(v)exp(\lambda_{i}(v)t)\sum_{j=0}^{n} e_{ij}(v) \right)^{1/R(v)}}.$$

It is clear that the above limit is equal to zero if the maximum of the real parts of the eigenvalues of the characteristic equation (denoted by$\mathfrak{R}$) divided by the corresponding value of $R(v)$ is higher for strategy $v$ than for strategy$w$. Since all coefficients of the characteristic equation are continuous function(al)s of$v$, its solution remains continuous.

Thus if max[${\mathfrak{R(}\lambda}_{i}(v))/R(v))]>max[{\mathfrak{R(}\lambda}_{i}(w))/R(w))]$, then there exist some neighbourhoods $O_{\varepsilon}\left( v^{'} \right)$and$O_{\varepsilon}\left( w^{'} \right)$, in which this inequality holds. In this case, the limit of $\eta(v,t)/\eta(w,t)$ is uniform in$O_{\varepsilon}\left( v^{'} \right)$and$O_{\varepsilon}\left( w^{'} \right)$. Therefore,$v\succ w$.

In other words, the function(al) $J\left( v \right)=\max_{i} \frac{{\mathfrak{R(}\lambda}_{i}(v))}{R(v)}$ reflects the introduced order of preference and can be considered as an evolutionary fitness. This proves Theorem 4.

1. Special case of a structured population.

Consider a particular case of the population structuring where only the last developmental stage can produce offspring, thus,$b_{i}=0$, $i=1,2,\ldots n-1$ and$b_{n}>0$. The characteristic equation derived in Theorem 4 can be substantially simplified. The first $n-1$ eigenvalues are given by $\lambda=a_{i}$, $i=1,2,\ldots n-1$. The other eigenvalues are given by the following transcendental equation.

$\lambda=b_{n}exp\left( -\sum_{k=0}^{n-1} a_{k}\left( \tau_{k+1}-\tau_{k} \right) \right)\left[ exp\left( -\tau_{n}\lambda\right)-exp\left( -\tau_{n+1}\lambda-a_{n}(\tau_{n+1}\left( v \right)-\tau_{n}\left( v \right)) \right) \right]-a_{n}$

Finally, the equation for the evolutionary fitness *J*(*v*) is given by the following implicit equation

$J\left( v \right)=\frac{b_{n}\left( v \right)}{R\left( v \right)}exp\left( -\sum_{k=0}^{n-1} a_{k}\left( v \right)\left( \tau_{k+1}(v)-\tau_{k}(v) \right) \right)\left[ exp\left( -\tau_{n}\left( v \right)J\left( v \right)R(v) \right)-exp\left( -\tau_{n+1}\left( v \right)J\left( v \right)R\left( v \right)-a_{n}(v \right)\left( \tau_{n+1}(v)-\tau_{n}(v) \right) \right]-\frac{a_{n}(v)}{R(v)}$ .

We will use the above implicit equation for fitness in applications to modelling vertical migration of zooplankton.

**SM2**

Here we provide key details on the collection of zooplankton samples and the methods of estimation of the spatially averaged depth of zooplankton migration. We start this section with a brief generic description of the considered ecosystem.

The Black Sea is a deep basin with a narrow active layer. It is characterized by permanent anoxia below the sharp pycnocline which is associated with a sigma-theta potential density higher than 14.3. The depth of the permanent pycnocline varies at temporal and spatial scales within a 100-150 m depth interval, depending on the water dynamics: as a rule, it is shallow in the central part of the basin, and deepens near the continental slope (Vinogradov and Nalbandov, 1990). It was reported that in the daytime, migrating herbivorous zooplankton usually aggregate in the suboxic layer above the depth of sigma theta 15.7 with oxygen concentration of 0.4 ml/l (Arashkevich et al., 2013).

The stations of zooplankton collection in this study were positioned over the upper part of the continental slope (44°29.44′ N, 37°58.38’ E), and three stations (in March 2009, April 2008, and December 2010) in the deep basin (43°53.53’ N, 37°30.37’ E). The geographic locations of the stations are shown in the map in Fig.S1.


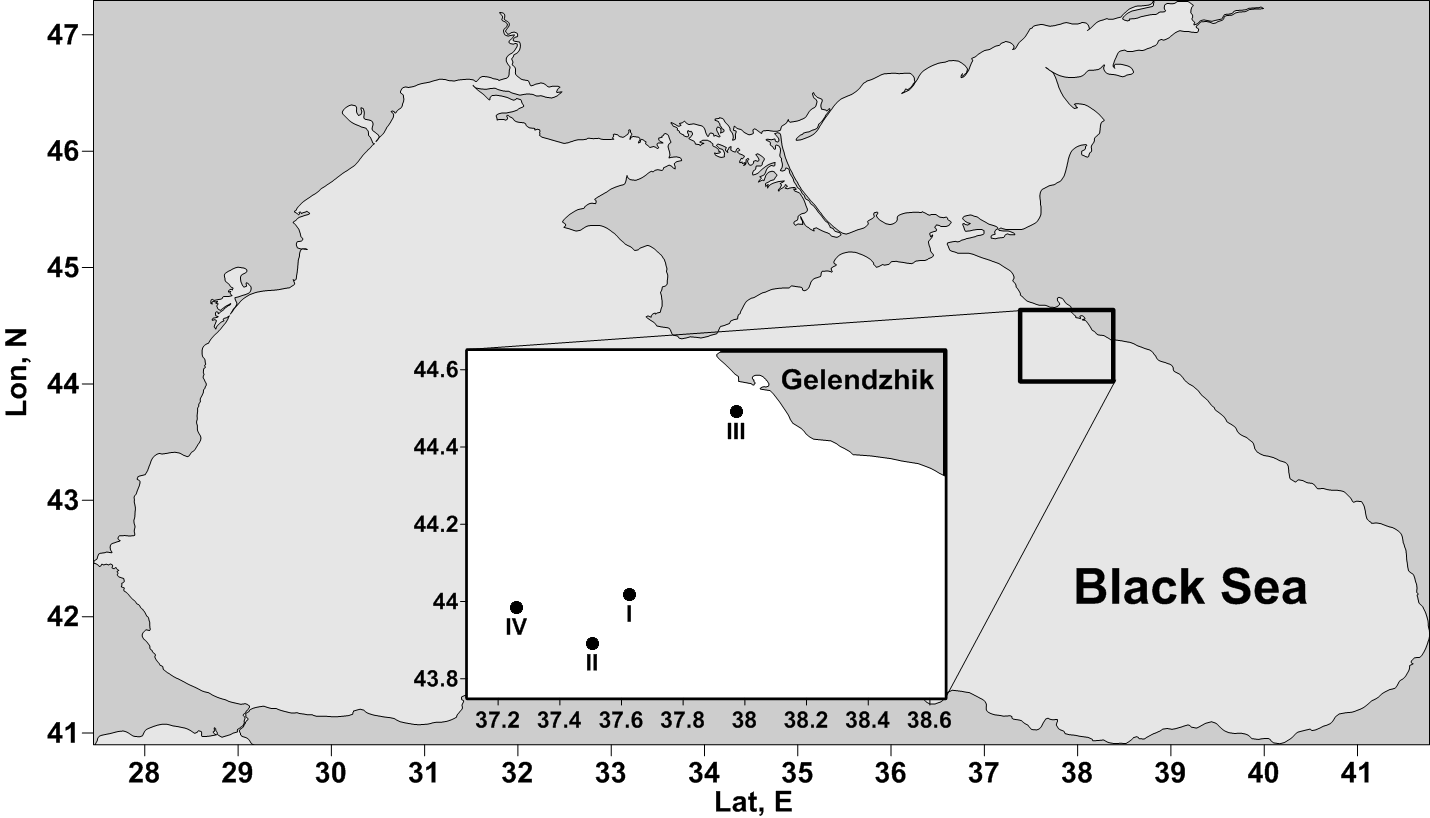


Fig.S1. Geographic location of the four stations in the Black Sea where samples were taken.

Vertical hauls of nets were performed at discrete layers within the whole oxic zone. Depth strata were chosen based on CTD proﬁles (obtained with a *SeaBird SBE 55в* CTD instrument) to sample the upper mixed layer, thermocline layer, the cold intermediate layer and two layers in the suboxic zone, from the depth of sigma theta between 15.9 and 15.7 and between 15.7 and 15.4.

For each station, zooplankton samples were taken near midday and near midnight (local standardized time) with a Juday net (mesh size 180μm, mouth area 0.1 m^2^). Net samples were preserved in a 4% formaldehyde-seawater solution until enumeration. The volume of filtered sea water was estimated based on the area of the net mouth and the length of the released wire. Analysis of zooplankton samples (to determine different species and distinguish separate developmental stages within the same species) was performed under a dissecting stereo-microscope.

For the measurement of chlorophyll-a concentration, water samples were collected at 5-6 selected depths according to the CTD profile and the *in situ* fluorometer readings. Water was filtered on GFF filters and chlorophyll-*a* concentration was measured fluorometrically in acetone extracts. We found that chlorophyll profiles show high seasonal and within-year variation. The averaged profile of chlorophyll across all observation is shown in Fig.2D of the main text. We do not show here the individual chlorophyll a profiles for each station for brevity.

The mean depth (*Z_m_*) and the standard deviation (*Z_s_*) of the vertical distribution of zooplankton species at the time of sampling were calculated according to equations by Grenvald et al (2016) modified from Sørnes et al. (2007):

$$Z_{m}= \sum_{j=1}^{n} w_{j}z_{j},$$

$Z_{s}=\sqrt{\sum_{j=1}^{n} w_{j}z_{j}^{2}-Z_{m}^{2}}$,

$w_{j}={d_{j}f_{j}}/{\sum_{j=1}^{n} d_{j}}f_{j}$,

where *n* is the number of depth intervals, *d_j_* = lower sample–upper sample depth (m) of sample interval *j*, *z_j_* is the midstrata (m) of sample interval *j*, *f_j_* is the density of individuals (ind. m^-3^) observed in the vertical interval *j* and *w_j_* is the relative abundance at depth stratum *j* taking into account the range of the vertical interval.

Examples of computation of the mean depths of zooplankton distribution (for the two most abundant species *Calanus euxinus* and *Pseudocalanus elongatus*) across 24 hours (with 3h sampling intervals) on June 21, 2011 are presented in Fig.S2 and Fig.S3 below. These data set correspond to the graphs shown in Figs.1A,B of the main text. We should also note that we do not show here the data on the diapausing subpopulations (stage CV) which do not perform regular diel vertical migration, but permanently stay near the boundary of the oxic zone.

Fig. S2. Diel variation of vertical distribution of copepod *Calanus euxinus* on June 21, 2011 (NE Black Sea). The panels correspond to the following developmental stages (A) – females - CVI, (B) – CV, (C) –(CIV), (D) – CI-III. The corresponding mean depth (*Z_m_*) and the standard deviation (*Z_s_*) are shown in the right column.

Fig. S3. Diel variation of vertical distribution of copepod *Pseudocalanus elongatus* on June 21, 2011 (NE Black Sea). The panels correspond to the following developmental stages (A) – females - CVI, (B) – CV, (C) –(CIV), (D) – CI-III. The corresponding mean depth (*Z_m_*) and the standard deviation (*Z_s_*) are shown in the right column.

**References for SM2**

Arashkevich E, Ostrovskii A, Solovyev V (2013) Observations of water column habitats by combining acoustic backscatter data and zooplankton sampling in the NE Black Sea. 40th CIESM Congress Proceedings, 103-104 Rapp. Comm. int. Mer Médit., 40, p 722.

Grenvald J.C., Callesen T.A., Daase M., Hobbs L., Darnis G., Renaud P.E., Cottier F., Nielsen T.G., Berge J. (2016). Plankton community composition and vertical migration during polar night in Kongsfjorden. Polar Biol 39:1879–1895

Ostrovskii AG, Zatsepin AG (2016) Intense ventilation of the Black Sea pycnocline due to vertical turbulent exchange in the Rim Current area. Deep-Sea Research I 116:1–13.

Sørnes TA, Aksnes DL, Båmstedt U, Youngbluth MJ (2007) Causes for mass occurrences of the jellyfish, Periphylla periphylla; a hypothesis that involves optically conditioned retention. J Plankton Res 29:157–167

Vinogradov ME, Nalbandov YuR (1990) Influence of water density on the distribution of physical, chemical, and biological characteristics of the Black Sea pelagic ecosystem. Oceanology 30(5):769–777

**SM3**

Zooplankton fitness may be largely affected by abiotic factors, in particular the temperature and the oxygen level. Here we present empirical data on both temperature and sigma-theta profiles for the given ecosystem across seasons.

Vertical proﬁles of temperature, salinity, density and fluorescence were obtained with a *SeaBird SBE 55в* CTD instrument equipped with a fluorometer prior to mesozooplankton sampling. Table TS1 shows details on locations, the dates and the numbers of sampled layers as well as the vertical positions of the critical depths of the temperature and sigma-theta profiles.

Table TS1. Date, location, and characterization of stations. In this table, * signifies sea surface temperature of 9.5 ℃ and ** denotes the sea surface temperature = 11 ℃.

| Date | Location | Coordinates | | Depth of isotherm  12 ℃ (m) | Depth of sigma-theta=15.7 (m) | Number  of sampled layers |
| --- | --- | --- | --- | --- | --- | --- |
|  |  | Latitude (N) | Longitude (E) |  |  |  |
| 29.03.2009 | I | 44°01.11’ | 37°37.61’ | * | 77 | 4 |
| 22.04.2008 | II | 43°53.53’ | 37°30.37’ | ** | 81 | 4 |
| 14.05.2013 | III | 44°29.44’ | 37°58.38’ | 11 | 149 | 5 |
| 21.6.2010 | III | 44°29.44’ | 37°58.38’ | 25 | 145 | 5 |
| 21.6.2011 | III | 44°29.44’ | 37°58.38’ | 15 | 147 | 6 |
| 17.07.2007 | III | 44°29.44’ | 37°58.38’ | 14 | 128 | 5 |
| 02.07.2014 | III | 44°29.44’ | 37°58.38’ | 31 | 132 | 5 |
| 30.09.2012 | III | 44°29.44’ | 37°58.38’ | 29 | 116 | 4 |
| 3.10.2012 | III | 44°29.44’ | 37°58.38’ | 27 | 108 | 4 |
| 5.10.2016 | III | 44°29.44’ | 37°58.38’ | 39 | 133 | 4 |
| 23.12.2010 | IV | 43°59.09’ | 37°15.64’ | 0 | 76 | 4 |

Fig.S4 shows seasonal variation of temperature profiles obtained with a *SeaBird SBE 55в* CTD-probe.

|  |
| --- |
|  |
| Fig.S4. Temperature profiles in the Northern-Eastern Black Sea observed in different seasons in 2007-2014. |

|  |
| --- |
|  |

Fig.S5 shows seasonal variation of potential density profiles measured across the seasons. Dashed lines indicate the potential density sigma-theta 14.7 (oxygen concentration ≈ 3 ml l^-1^) and 15.7 (oxygen concentration ≈ 0.4 ml l^-1^).

Finally, we show below an example of the empirical depth dependence of the basal and the total metabolism rates of and estimated for *Calanus euxinus* (adults CVI) for the density sigma-theta profile corresponding to station III measured on June 21, 2011. We fitted the data using the following analytical parameterisations: *m_Ab_*(*h*)=*m_A_*(tanh(-*σ_m_*(*h*-*h_m_*))+1)*/*2 and *M_A_*(*h*)=*M_A0_*(tanh(-*σ_M_*(*h*-*h_M_*))+1)*/*2 (see the main text for more detail). The non-linear fitting (PRISM7 software) gives the following estimates for parameters *m_A_*=0.144±0.001 *μl*O_2_/ind h; *σ_m_*=0.0480±0.004 1/m; *h_m_*=119±0.5m; *M_A0_*=0.335±0.003 *μl*O_2_/ind h; *σ_M_*=0.0458±0.005 1/m; *h_M_*=121.0±0.7m.

Fig. S6. The depth dependence of the metabolic rates (basal and total) for *Calanus euxinus* (stage CVI) estimated for the environmental conditions at station III on June 21, 2011.

**SM4**

Here we derive the analytical equations determining the optimal parameters of evolutionary fitness *J* given by equation (5) from the main text of the article. These equations are variational equations of selection for the considered model.

We firstly calculate the ages of transition between the stages which are determined by the weights. The equation of the weight growth is given by (4)

$\frac{dW}{dt}=r_{i}(v,W)$.

The considered three stages are characterized by the following ranges:

*i=Y*; *W* $\in$ [*W_1_*,*W_Y_*];

*i=J*, *W* $\in$ [*W_Y_*,*W_A_*];

*i=A*, *W*=*W_A_*=const.

We consider the piecewise linear structure of the trajectories of DVM (see the main text). In this case, for the growth rates (*i=Y,J*) we have

$r_{i}( v,W)=\left[ \varepsilon_{i}\frac{\alpha_{i}(W)P\left( H_{i0} \right)}{1+\alpha P\left( H_{i0} \right)}\left( t_{i0}+1-t_{i3} \right)-Metabolic\_costs\_1 \right]$,

where the first term describes the food consumed while at the shallowest depth. Furthermore,

$Metabolic\_costs\_1= \left( t_{i0}+1-t_{i3} \right)m_{i}(W)(tanh({-\sigma}_{m}\left( H_{i0}-h_{m} \right))+1)$/2

+$\left( t_{i2}-t_{i1} \right)m_{i}\left( W \right)(tanh({-\sigma}_{m}\left( H_{i1}-h_{m} \right))+1)$/2

+$\int_{t_{i0}}^{t_{i1}} m_{i}\left( W \right)(tanh({-\sigma}_{m}\left( c_{i0}\left( t-t_{i0} \right)+H_{i0}-h_{m} \right))+1)/2dt +$

$$+\int_{t_{i2}}^{t_{i3}} m_{i}\left( W \right)(tanh(-\sigma_{m}\left( -c_{i1}\left( t-t_{i2} \right)+H_{i1}-h_{m} \right))+1)/2dt +$$

$+\left( t_{i0}+1-t_{i3} \right)M_{i0}\left( W \right)(tanh({-\sigma}_{m}\left( H_{i0}-h_{m} \right))+1)$/2 +

$+\int_{t_{i2}}^{t_{i3}} M_{i0}\left( W \right)(tanh(-\sigma_{m}\left( -c_{i1}\left( t-t_{i2} \right)+H_{i1}-h_{m} \right)+1)/2dt$

The first four terms are the basic metabolic costs during the four phases. The last two terms describe the metabolic cost spent on active movement while feeding and while ascending, but not while descending or staying without feeding.

The dependence of the coefficients on the weight *W* is given by (12). We can calculate the integrals in the above expressions for the metabolic costs. We obtain after simplification

$$\int_{t_{i0}}^{t_{i1}} m_{i}\left( W \right)(\tanh{(-\sigma}_{m}\left( c_{i0}\left( t-t_{i0} \right)+H_{i0}-h_{m} \right))+1)/2\mathrm{dt} =$$

$$=\frac{m_{i}\left( W \right)}{2}(\frac{1}{{-\sigma}_{m}c_{i0}}\ln\frac{\cosh{(-\sigma}_{m}c_{i0}t_{i1}+\sigma_{m}c_{i0}t_{i0}-\sigma_{m}H_{i0}+{\sigma_{m}h}_{m})}{\cosh({-\sigma}_{m}H_{i0}+{\sigma_{m}h}_{m})}+\left( t_{i1}-t_{i0} \right))$$

$$\int_{t_{i2}}^{t_{i3}} m_{i}(W)tanh(-\sigma_{m}\left( \left( -c_{i1}\left( t-t_{i2} \right)+H_{i1} \right)-h_{m} \right)+1)/2dt=$$

$$=\frac{m_{i}\left( W \right)}{2}(\frac{1}{\sigma_{m}c_{i1}}\ln\frac{\cosh{(\sigma}_{m}c_{i1}t_{i3}-\sigma_{m}c_{i1}t_{i2}-\sigma_{m}H_{i1}+{\sigma_{m}h}_{m})}{\cosh(-\sigma_{m}H_{i1}+{\sigma_{m}h}_{m})}+\left( t_{i3}-t_{i2} \right))$$

$\int_{t_{i2}}^{t_{i3}} M_{i0}(W)\tanh({-\sigma}_{m}\left( \left( -c_{i1}\left( t-t_{i2} \right)+H_{i1} \right)-h_{m} \right)+1)/2\mathrm{dt}$=

$$=\frac{M_{i0}\left( W \right)}{2}\left( \frac{1}{\sigma_{m}c_{i1}}\ln\frac{\cosh{(\sigma}_{m}c_{i1}t_{i3}-\sigma_{m}c_{i1}t_{i2}-\sigma_{m}H_{i1}+{\sigma_{m}h}_{m})}{\cosh\left( {-\sigma}_{m}H_{i1}+{\sigma_{m}h}_{m} \right)}+\left( t_{i3}-t_{i2} \right) \right).$$

Using the above formulas, $r_{i}(v, W)$ can be expressed as

$$r_{i}\left( v, W \right){=W}^{0.8}(\frac{\varepsilon_{i}CP\left( H_{i0} \right)}{1+\alpha P\left( H_{i0} \right)}\left( t_{i0}+1-t_{i3} \right)-\frac{\left( t_{i0}+1-t_{i3} \right){(C}_{1}+C_{2})\left( \tanh\left( -\sigma_{m}\left( H_{i0}-h_{m} \right) \right)+1 \right)}{2}$$

-$\left( t_{i2}-t_{i1} \right)C_{1}(tanh({-\sigma}_{m}\left( H_{i1}-h_{m} \right))+1)$/2

$$-\frac{C_{1}}{2}\left( \frac{-1}{\sigma_{m}c_{i0}}\ln\frac{\cosh{(-\sigma}_{m}c_{i0}t_{i1}+\sigma_{m}c_{i0}t_{i0}-\sigma_{m}H_{i0}+{\sigma_{m}h}_{m})}{\cosh\left( -\sigma_{m}H_{i0}+{\sigma_{m}h}_{m} \right)}+\left( t_{i1}-t_{i0} \right) \right)$$

$$-\frac{C_{1}+C_{2}}{2}(\frac{1}{\sigma_{m}c_{i1}}\ln\frac{\cosh{(\sigma}_{m}c_{i1}t_{i3}-\sigma_{m}c_{i1}t_{i2}-\sigma_{m}H_{i1}+{\sigma_{m}h}_{m})}{\cosh\left( -\sigma_{m}H_{i1}+{\sigma_{m}h}_{m} \right)}+\left( t_{i3}-t_{i2} \right))).$$

We denote

$$Q_{i}=(\frac{\varepsilon_{i}CP\left( H_{i0} \right)}{1+\alpha P\left( H_{i0} \right)}\left( t_{i0}+1-t_{i3} \right)-\frac{\left( t_{i0}+1-t_{i3} \right){(C}_{1}+C_{2})\left( \tanh\left( {-\sigma}_{m}\left( H_{i0}-h_{m} \right) \right)+1 \right)}{2}$$

-$\left( t_{i2}-t_{i1} \right)C_{1}(tanh({-\sigma}_{m}\left( H_{i1}-h_{m} \right))+1)$/2

$$-\frac{C_{1}}{2}\left( \frac{-1}{\sigma_{m}c_{i0}}\ln\frac{\cosh{(-\sigma}_{m}c_{i0}t_{i1}+\sigma_{m}c_{i0}t_{i0}-\sigma_{m}H_{i0}+{\sigma_{m}h}_{m})}{\cosh\left( {-\sigma}_{m}H_{i0}+{\sigma_{m}h}_{m} \right)}+\left( t_{i1}-t_{i0} \right) \right)$$

$$-\frac{C_{1}+C_{2}}{2}(\frac{1}{\sigma_{m}c_{i1}}\ln\frac{\cosh{(\sigma}_{m}c_{i1}t_{i3}-\sigma_{m}c_{i1}t_{i2}-\sigma_{m}H_{i1}-{\sigma_{m}h}_{m})}{\cosh\left( -\sigma_{m}H_{i1}+{\sigma_{m}h}_{m} \right)}+\left( t_{i3}-t_{i2} \right))).$$

We should stress that *Q*_i_ does not depend on the weight *W*.

To determine the age at which each stage starts we will integrate the growth equation which gives

$\frac{dW}{dt}=Q_{i}W^{0.8}$ , $W^{-0.8}dW=Q_{i}dt$ , $W^{0.2}=Q_{i}t+const$.

We obtain

$${{W=(Q}_{i}t+const)}^{-0.2} .$$

From the initial conditions we have

${W_{1}=(const)}^{-0.2}$ ; ${{W_{1}}^{0.2}=const}$

$${{W_{Y}=(Q}_{Y}\tau_{1}+{W_{1}}^{0.2})}^{-0.2}$$

$$\frac{{W_{Y}}^{0.2}-{W_{1}}^{0.2}}{Q_{Y}}=\tau_{1}$$

$${{W_{A}=(Q}_{J}t-Q_{J}\tau_{1}+Q_{Y}\tau_{1}+{W_{1}}^{0.2})}^{-0.2}={{(Q}_{J}t-Q_{J}\tau_{1}+{W_{Y}}^{0.2})}^{-0.2}={{(Q}_{J}t-Q_{J}\frac{{W_{Y}}^{0.2}-{W_{1}}^{0.2}}{Q_{Y}}+{W_{Y}}^{0.2})}^{-0.2}$$

$${{W_{A}=(Q}_{J}\tau_{2}-Q_{J}\tau_{1}+{W_{Y}}^{0.2})}^{-0.2}$$

$$\frac{{W_{A}}^{0.2}-{W_{Y}}^{0.2}+Q_{J}\tau_{1}}{Q_{J}}=\tau_{2}$$

$$\frac{{W_{A}}^{0.2}-{W_{Y}}^{0.2}}{Q_{J}}+\frac{{W_{Y}}^{0.2}-{W_{1}}^{0.2}}{Q_{Y}}=\tau_{2}$$

In the piecewise linear structure of DVM (see the main text), for fixed maximal and minimal depths of migration, only two times of switching are independent. Without losing generality we can consider *t_i1_* and *t_i2_* as independent parameters we assume that c_i0_ = c_i1_. We obtain *t_i0_−t_i3_= t_i1_*− (*H_i1_− H_i0_*)/ *c_i0_ − t_i2_*− (*H_i1_− H_i0_*)/ c*_i1_*= *t_i1_−t_i2_*−2 (*H_i1_− H_i0_*)/ c_i0_.

We can re-arrange and simplify the obtained expressions for $Q_{i}$

$Q_{i}=\left( t_{i1}-t_{i2}-2\frac{H_{i1}-H_{i0}}{c_{i0}}+1 \right)\left( \frac{\varepsilon_{i}CP\left( H_{i0} \right)}{1+\alpha P\left( H_{i0} \right)}-\frac{{(C}_{1}+C_{2})\left( \tanh\left( -\sigma_{m}\left( H_{i0}-h_{m} \right) \right)+1 \right)}{2} \right)$

$-\left( t_{i2}-t_{i1} \right)C_{1}(tanh(-\sigma_{m}\left( H_{i1}-h_{m} \right))+1)$/2

$-\left( C_{1}+\frac{C_{2}}{2} \right)\left( \frac{-1}{\sigma_{m}c_{i0}}\ln\frac{\cosh{(-\sigma}_{m}H_{i1}+{\sigma_{m}h}_{m})}{\cosh\left( {-\sigma}_{m}H_{i0}+{\sigma_{m}h}_{m} \right)}+\frac{H_{i1}-H_{i0}}{c_{i0}} \right)=Q_{i}\left( t_{i2}-t_{i1} \right).$

In other words, *Q*_i_ is a linear function of the difference $\left( t_{i2}-t_{i1} \right)$ .From here we can easily derive the following identity for the derivatives

$$\frac{\partial Q_{i}}{\partial t_{i2}}=-\frac{\partial Q_{i}}{\partial t_{i1}}.$$

Now we can calculate the mortality rates $a_{i}$. After simplification we obtain

$a_{i}=\int_{0}^{1} (S_{i}\gamma_{i}\left( \tanh\left( -\sigma\left( h_{i}\left( t \right)-h_{p} \right) \right)+1 \right)(-\cos2\pi t+1)/4 +A_{i}(h_{i}\left( t \right))+\gamma_{i0})dt$=

$$=\frac{\gamma_{i}\left( \tanh\left( -\sigma\left( H_{i1}-h_{p} \right) \right)+1 \right)}{4}(\frac{-1}{2\pi}\left( \sin2\pi t_{i2}-\sin2\pi t_{i1} \right)+\left( t_{i2}-t_{i1} \right))$$

$$+\left( t_{i1}-t_{i2}-2\frac{H_{i1}-H_{i0}}{c_{i0}}+1 \right)(\delta_{u}\left( \tanh\left( {-\sigma}_{u}\left( H_{i0}-h_{u} \right) \right)+1 \right)+\delta_{d}\left( \tanh\left( \sigma_{d}\left( H_{i0}-h_{u} \right) \right)+1 \right))$$

+$\left( t_{i2}-t_{i1} \right)(\delta_{u}\left( \tanh\left( {-\sigma}_{u}\left( H_{i1}-h_{u} \right) \right)+1 \right)+\delta_{d}\left( \tanh\left( \sigma_{d}\left( H_{i1}-h_{u} \right) \right)+1 \right))$+

$$+\delta_{u}\frac{-1}{\sigma_{u}c_{i0}}\ln\frac{\tanh\left( {-\sigma}_{u}\left( H_{i1}-h_{u} \right) \right)}{\tanh\left( -\sigma_{u}\left( H_{i0}-h_{u} \right) \right)}-\delta_{d}\frac{-1}{\sigma_{d}c_{i0}}\ln\frac{\tanh\left( -\sigma_{d}\left( H_{i1}-h_{d} \right) \right)}{\tanh\left( {-\sigma}_{d}\left( H_{i0}-h_{d} \right) \right)}$$

$${+(\delta}_{u}+\delta_{d})\left( t_{i1}-t_{i0} \right)$$

$+\delta_{u}\frac{1}{\sigma_{u}c_{i1}}\ln\frac{\tanh\left( {-\sigma}_{u}\left( H_{i0}+h_{u} \right) \right)}{\tanh\left( {-\sigma}_{u}\left( H_{i1}+h_{u} \right) \right)}-\delta_{d}\frac{1}{\sigma_{d}c_{i1}}\ln\frac{\tanh\left( {-\sigma}_{d}\left( H_{i0}-h_{d} \right) \right)}{\tanh\left( {-\sigma}_{d}\left( H_{i1}-h_{d} \right) \right)}+{(\delta}_{u}+\delta_{d})\left( t_{i3}-t_{i2} \right)+\gamma_{i0}$ . Hence,

$$\frac{\partial a_{i}}{\partial t_{i1}}=\frac{\gamma_{i}\left( \tanh\left( -\sigma\left( H_{i1}-h_{p} \right) \right)+1 \right)}{4}(\left( \cos2\pi t_{i1} \right)-1)$$

$$(\delta_{u}\left( \tanh\left( -\sigma_{u}\left( H_{i0}-h_{u} \right) \right)+1 \right)+\delta_{d}\left( \tanh\left( \sigma_{d}\left( H_{i0}-h_{u} \right) \right)+1 \right))$$

$$-\left( \delta_{u}\left( \tanh\left( {-\sigma}_{u}\left( H_{i1}-h_{u} \right) \right)+1 \right)+\delta_{d}\left( \tanh\left( \sigma_{d}\left( H_{i1}-h_{u} \right) \right)+1 \right) \right).$$

Moreover,

$$\frac{\partial a_{i}}{\partial t_{i2}}=\frac{\gamma_{i}\left( \tanh\left( -\sigma\left( H_{i1}+h_{p} \right) \right)+1 \right)}{4}(\left( -cos 2\pi t_{i2} \right)+1)$$

$\left( -1 \right)(\delta_{u}\left( \tanh\left( {-\sigma}_{u}\left( H_{i0}-h_{u} \right) \right)+1 \right)+\delta_{d}\left( \tanh\left( \sigma_{d}\left( H_{i0}-h_{u} \right) \right)+1 \right))$+

+$(\delta_{u}\left( \tanh\left( -\sigma_{u}\left( H_{i1}-h_{u} \right) \right)+1 \right)+\delta_{d}\left( \tanh\left( \sigma_{d}\left( H_{i1}-h_{u} \right) \right)+1 \right))$.

Now we maximize fitness *J* given by (7) by differentiating with respect to parameters *J*. Note that $\tau_{3}-\tau_{2}=T_{0}=const$. Computation of ∂*J*/∂*t _Y1_*=0 gives (after some simplification)

$$0=\exp\left( -\tau_{2}JR \right)(-\frac{\partial a_{0}}{\partial t_{Y1}}\tau_{1}-a_{0}\frac{\partial\tau_{1}}{\partial t_{Y1}}-a_{1}\left( \frac{\partial\tau_{2}}{\partial t_{Y1}}-\frac{\partial\tau_{1}}{\partial t_{Y1}} \right)-\frac{\partial\tau_{2}}{\partial t_{Y1}}JR)-\exp\left( -a_{2}\left( \tau_{3}-\tau_{2} \right) \right)(-\frac{\partial a_{0}}{\partial t_{Y1}}\tau_{1}-a_{0}\frac{\partial\tau_{1}}{\partial t_{Y1}}-a_{1}\left( \frac{\partial\tau_{2}}{\partial t_{Y1}}-\frac{\partial\tau_{1}}{\partial t_{Y1}} \right))$$

We find the derivatives $\frac{\partial\tau_{1}}{\partial t_{Y1}}$ and $\frac{\partial\tau_{2}}{\partial t_{Y1}}$

$$\frac{\partial\tau_{1}}{\partial t_{Y1}}=-\frac{{W_{Y}}^{0.2}-{W_{0}}^{0.2}}{{Q_{Y}}^{2}}\frac{\partial Q_{Y}}{\partial t_{Y1}}$$

$$\frac{\partial\tau_{2}}{\partial t_{Y1}}=-\frac{{W_{Y}}^{0.2}-{W_{0}}^{0.2}}{{Q_{Y}}^{2}}\frac{\partial Q_{Y}}{\partial t_{Y1}}=\frac{\partial\tau_{1}}{\partial t_{Y1}}.$$

Then we have

$$0=\exp\left( -\tau_{2}JR \right)\left( -\frac{\partial a_{0}}{\partial t_{Y1}}\tau_{1}-a_{0}\frac{\partial\tau_{1}}{\partial t_{Y1}}-\frac{\partial\tau_{1}}{\partial t_{Y1}}JR \right)-\exp\left( -a_{2}T_{0} \right)\left( -\frac{\partial a_{0}}{\partial t_{Y1}}\tau_{1}-a_{0}\frac{\partial\tau_{1}}{\partial t_{Y1}} \right).$$

Hence

$$(\exp\left( -\tau_{2}JR \right)-\exp\left( -a_{2}T \right))\frac{\partial a_{0}}{\partial t_{Y1}}\tau_{1}=(-\exp\left( -\tau_{2}JR \right)a_{0}-\exp\left( -\tau_{2}JR \right)JR+\exp\left( -a_{2}T_{0} \right)a_{0})\frac{\partial\tau_{1}}{\partial t_{Y1}}$$

Computation of ∂J/∂t _Y2_=0 gives (after some simplification)

$$0=\frac{b}{R}\exp\left( -a_{0}\tau_{1}-a_{1}\left( \tau_{2}-\tau_{1} \right)-\tau_{2}JR \right)(-\frac{\partial a_{0}}{\partial t_{Y2}}\tau_{1}-a_{0}\frac{\partial\tau_{1}}{\partial t_{Y1}}-a_{1}\left( \frac{\partial\tau_{2}}{\partial t_{Y2}}-\frac{\partial\tau_{1}}{\partial t_{Y2}} \right)-\frac{\partial\tau_{2}}{\partial t_{Y1}}JR)-\frac{b}{R}\exp\left( -a_{0}\tau_{1}-a_{1}\left( \tau_{2}-\tau_{1} \right)-a_{2}\left( \tau_{3}-\tau_{2} \right) \right)(-\frac{\partial a_{0}}{\partial t_{Y2}}\tau_{1}-a_{0}\frac{\partial\tau_{1}}{\partial t_{Y2}}-a_{1}\left( \frac{\partial\tau_{2}}{\partial t_{Y2}}-\frac{\partial\tau_{1}}{\partial t_{Y2}} \right))$$

We find the derivatives$\frac{\partial\tau_{1}}{\partial t_{Y2}}$, $\frac{\partial\tau_{2}}{\partial t_{Y2}}$

$$\frac{\partial\tau_{1}}{\partial t_{Y2}}=-\frac{{W_{Y}}^{0.2}-{W_{0}}^{0.2}}{{Q_{Y}}^{2}}\frac{\partial Q_{Y}}{\partial t_{Y2}},$$

$$\frac{\partial\tau_{2}}{\partial t_{Y2}}=-\frac{{W_{Y}}^{0.2}-{W_{0}}^{0.2}}{{Q_{Y}}^{2}}\frac{\partial Q_{Y}}{\partial t_{Y2}}=\frac{\partial\tau_{1}}{\partial t_{Y2}}.$$

Then we have

$$0=\exp\left( -\tau_{2}JR \right)\left( -\frac{\partial a_{0}}{\partial t_{Y2}}\tau_{1}-a_{0}\frac{\partial\tau_{1}}{\partial t_{Y2}}-\frac{\partial\tau_{1}}{\partial t_{Y2}}JR \right)-\exp\left( -a_{2}T \right)\left( -\frac{\partial a_{0}}{\partial t_{Y2}}\tau_{1}-a_{0}\frac{\partial\tau_{1}}{\partial t_{Y2}} \right).$$

Hence

$$\left( \exp\left( -\tau_{2}JR \right)-\exp\left( -a_{2}T_{0} \right) \right)\frac{\partial a_{0}}{\partial t_{Y2}}\tau_{1}=\left( -\exp\left( -\tau_{2}JR \right)a_{0}-\exp\left( -\tau_{2}JR \right)JR+\exp\left( -a_{2}T_{0} \right)a_{0} \right)\frac{\partial\tau_{1}}{\partial t_{Y2}}.$$

Using the identity (see the proof above)

$$\frac{\partial Q_{i}}{\partial t_{i2}}=-\frac{\partial Q_{i}}{\partial t_{i1}}.$$

We have

$$\frac{\partial\tau_{1}}{\partial t_{Y2}}=-\frac{\partial\tau_{1}}{\partial t_{Y1}}.$$

From the both above identities we have

$$\frac{\partial a_{0}}{\partial t_{Y2}}=-\frac{\partial a_{0}}{\partial t_{Y1}}.$$

We derive that

$$\frac{\gamma_{Y}\left( \tanh\left( -\sigma\left( H_{Y1}-h_{p} \right) \right)+1 \right)}{4}\left( \cos2\pi t_{Y1} \right)=\frac{\gamma_{Y}\left( \tanh\left( -\sigma\left( H_{Y1}-h_{p} \right) \right)+1 \right)}{4}\left( \cos2\pi t_{Y2} \right).$$

Hence $\cos(2\pi t_{Y1})=\cos(2\pi t_{Y2})$

We consider the same period of the given periodic function. In this case we have

$$t_{Y2}-\frac{1}{2}=\frac{1}{2}-t_{Y1}.$$

This proves the symmetry of the switching times $t_{Y1}$ and $t_{Y2}$ with respect to *t*=0.5, i.e. midnight. The symmetry of the other stages can be proved in a similar way.
